# GLP-1RAs in type 2 diabetes: mechanisms that underlie cardiovascular effects and overview of cardiovascular outcome data

**DOI:** 10.1186/s12933-018-0800-2

**Published:** 2018-12-13

**Authors:** Andrei C. Sposito, Otávio Berwanger, Luiz Sérgio F. de Carvalho, José Francisco Kerr Saraiva

**Affiliations:** 1 Atherosclerosis and Vascular Biology Laboratory (AtheroLab), Cardiology Division, Faculty of Medical Sciences, State University of Campinas (Unicamp), 13084-971 Campinas, Sao Paulo Brazil; 2 Academic Research Organization (ARO), Albert Einstein Hospital, Av. Albert Einstein 627, Sao Paulo, SP 05651-901 Brazil; 3 Cardiology Division, Pontifical Catholic University of Campinas Medicine School, Rua Engenheiro Carlos Stevenson 560, Campinas, Sao Paulo 13092-132 Brazil

**Keywords:** Cardiovascular outcomes, Diabetes, GLP-1 receptor agonist, Obesity

## Abstract

Patients with type 2 diabetes (T2DM) have a substantial risk of developing cardiovascular disease. The strong connection between the severity of hyperglycaemia, metabolic changes secondary to T2DM and vascular damage increases the risk of macrovascular complications. There is a challenging demand for the development of drugs that control hyperglycaemia and influence other metabolic risk factors to improve cardiovascular outcomes such as cardiovascular death, nonfatal myocardial infarction, nonfatal stroke, hospitalization for unstable angina and heart failure (major adverse cardiovascular events). In recent years, introduction of the new drug class of glucagon-like peptide-1 receptor agonists (GLP-1RAs) has changed the treatment landscape as GLP-1RAs have become well-established therapies in T2DM. The benefits of GLP-1RAs are derived from their pleiotropic effects, which include appetite control, glucose-dependent secretion of insulin and inhibition of glucagon secretion. Importantly, their beneficial effects extend to the cardiovascular system. Large clinical trials have evaluated the cardiovascular effects of GLP-1RAs in patients with T2DM and elevated risk of cardiovascular disease and the results are very promising. However, important aspects still require elucidation, such as the specific mechanisms involved in the cardioprotective effects of these drugs. Careful interpretation is necessary because of the heterogeneity across the trials concerning the definition of cardiovascular risk or cardiovascular disease, baseline characteristics, routine care and event rates. The aim of this review is to describe the main clinical aspects of the GLP-1RAs, compare them using data from both the mechanistic and randomized controlled trials and discuss potential reasons for improved cardiovascular outcomes observed in these trials. This review may help clinicians to decide which treatment is most appropriate in reducing cardiovascular risk in patients with T2DM.

## Background

The prevalence of type 2 diabetes (T2DM) has increased and approximately 425 million adults aged 20–79 years are estimated to have diabetes worldwide. If this trend continues, 629 million individuals will have diabetes by 2045 [1]. Genetic susceptibility, ageing and obesity are contributing to the increase in T2DM prevalence [2]. T2DM is mainly caused by a combination of insulin resistance, functional failure of pancreatic beta cells and excessive or inappropriate glucagon secretion [3]. T2DM is currently considered a systemic disease in which dysfunction in multiple organs and tissues contribute to hyperglycaemia [4].

Diabetes is associated with both micro- and macrovascular complications. Long-term damage to the microvasculature causes nephropathy, retinopathy and neuropathy [5]. Macrovascular complications are mainly represented by atherosclerotic ndisease. Disturbances of glucose levels caused by obesity-related insulin resistance or impaired insulin secretion cause endothelial and smooth muscle cell dysfunction [6–9]. Unfortunately, most patients have already developed vascular complications by the time they are diagnosed with T2DM. Comorbidities such as obesity, hypertension, and dyslipidaemia are important contributors to the increased risk of cardiovascular disease (CVD) in patients with T2DM. However, unlike non-diabetic patients, people with T2DM have an increased risk of CVD that is additional to and independent of conventional risk factors [10, 11]. As a result of the progressive nature of macrovascular and myocardial disease, T2DM patients have a risk of cardiovascular death or non-fatal atherothrombotic events similar to or higher than that of patients who had a previous myocardial infarction (MI) [12]. In the last two decades, several studies have demonstrated reductions in the risk of cardiovascular outcomes and mortality in patients with T2DM with improved glucose and blood pressure control and cholesterol-lowering therapies [13–15]. Nevertheless, macrovascular disease remains the most common cause of death in T2DM patients [16] and new diabetes therapies are highly desired, especially if they can offer cardiovascular benefits.

The strong link between T2DM and obesity is closely related to an impaired crosstalk between the brain and peripheral organs [17]. The “incretin [INtestine seCRETtion INsulin] effect” is defined as the increased stimulation of insulin secretion provoked by oral administration of glucose compared with intravenous administration at similar glucose levels. The incretin effect is mainly caused by the release of the gut hormones glucose-dependent insulinotropic polypeptide (GIP) and glucagon-like peptide-1 (GLP-1), which stimulate insulin secretion from pancreatic beta cells [18–20]. Incretins also regulate glucose concentrations, gut motility, lipid metabolism, immune function, appetite and body weight [21]. The demonstration that the actions of GLP-1 are reduced in T2DM [22] led to the development of incretin-based therapies, including GLP-1 receptor agonists (RAs) and dipeptidyl peptidase 4 (DPP-4) inhibitors. DPP-4 inhibitors block the degradation of GLP-1 and GIP, thus extending their insulinotropic effect. The glucose-lowering effects of bariatric surgery and the success of glucagon-like peptide-1 (GLP-1) receptor agonists (RAs) in the treatment of T2DM have confirmed the importance of the gut in the maintenance of neural and hormonal homeostasis in diabetes treatment [22].

This review focuses on clinical aspects of GLP-1RAs and their effects on cardiovascular outcomes such as cardiovascular death, non-fatal myocardial infarction (MI), non-fatal stroke, hospitalization for unstable angina and heart failure (major adverse cardiovascular events, MACE). We describe the large recent trials conducted in T2DM patients with high CVD risk, provide a critical overview of the resultant cardiovascular outcome data and discuss potential explanations for the improved cardiovascular outcomes reported so far.

## GLP-1, its receptor agonists and derived metabolites

GLP-1, a product of the glucagon gene, is a 30-amino-acid peptide produced in l-cells of the small intestine. Within minutes of food intake, the active form of GLP-1 is released into the circulation and activates specific G-protein coupled receptors. The GLP-1 receptor (GLP-1R) consists of 463 amino acids and contains eight hydrophobic domains. The homologues of the N-terminal extracellular hydrophobic domain expressed in tissues/organs such as the hypothalamus, lung, pancreatic islets, stomach, kidney, intestine and heart are highly preserved. GLP-1R activation leads to a rapid increase in the levels of cyclic adenosine monophosphate (AMP) and intracellular calcium followed by glucose-dependent insulin release. The GLP-1 hormone is rapidly inactivated by the enzyme dipeptidyl peptidase-4 (DPP-4), with a half-life of only 1–2 min [23, 24]. Amino acid modifications in the N-terminus and at certain positions in the C-terminus are also directly involved in the receptor interaction and resistance to DPP-4 inhibition, thus prolonging its effect and half-life [25].

GLP-1 circulates in many different forms, only some of which are biologically active. The active form of GLP-1 is GLP-1[7–36]amide, which represents a major secretory product. Once in the circulation, GLP-1[7–36]amide has a half-life of less than 2 min, being subject to rapid cleavage between positions 8 and 9 by the DPP-4 enzyme to its N-terminally truncated metabolite GLP-1[9–36]amide, which does not interact with the GLP-1R. Until recently, GLP-1[9–36]amide and other GLP-1 metabolites, including GLP-1[28–36]amide and GLP-1[32–36]amide, were considered to be metabolically inactive [26], at least at physiological concentrations. However, increasing evidence indicates that some of these metabolites have biological activity that may contribute to the pleiotropic effects of GLP-1 independently of the GLP-1 receptor [27, 28]. It has been reported that these metabolites have beneficial cardioprotective [29] and glucoregulatory actions when administered pharmacologically, such as reduction in oxidative stress in vascular tissues [30], protective actions on beta cells [31] and inhibition of gluconeogenesis and oxidative stress in hepatocytes [32].

GLP-1[7–36]amide and its metabolites have direct effects on cardiomyocyte viability, improving cardiac function, and vasodilation. Most importantly, the difference in the cardiovascular effects between metabolites is attracting attention [33]. For example, GLP-1[7–36]amide exerts cardiovascular effects through a GLP-1R-dependent pathway, whereas GLP-1[9–36]amide exerts its effects through a GLP-1R-independent pathway. Thus, these metabolites have a broad therapeutic potential, as targeting GLP-1R activation or GLP-1 degradation may have different cardiovascular consequences.

GLP-1 therapies have been established using two main approaches: DPP4-resistant GLP-1 agonists/GLP-1RA and DPP-4 inhibitors, both of which aim to prolong the life-time of circulating GLP-1[7–36]amide [34]. GLP-1RAs were developed to activate the GLP-1Rs and to be resistant or semi-resistant to inactivation by DPP-4, GLP-1RAs are classified according to their fundamental structure and pharmacokinetic properties. Some GLP-1RAs are structurally similar to native GLP-1 with some amino acid modifications to avoid degradation by the DPP-4 enzyme. Others were synthetically developed by replicating the structure of exendin-4, a naturally occurring peptide of 39 amino acids originally isolated from the saliva of the lizard *Heloderma suspectum*, which has GLP-1R-activating properties and is naturally resistant to degradation by the DPP-4 enzyme [25, 35].

GLP-1RAs are useful tools for the treatment of T2DM, especially for patients aiming weight loss or to hypoglycaemia [36]. GLP-1RAs target both fasting and postprandial glycaemia, in general by increasing insulin and decreasing glucagon levels. GLP-1 stimulates insulin release from the pancreatic islets in response to elevated glucose levels above fasting levels (glucose-dependent action), such as after a meal. When administered intravenously, GLP-1 does not decrease glucose below fasting levels [37, 38], therefore the GLP1-RAs are associated with a low incidence of hypoglycaemia. However, GLP-1RAs also decrease hepatic gluconeogenesis, improve insulin sensitivity and delay gastric emptying, potentially promoting central satiety and reducing overall caloric intake [39]. Furthermore, they enhance beta cell proliferation and have anti-apoptotic effects, inducing the biosynthesis of insulin [40, 41]. The most frequently reported adverse events associated with GLP-1RAs include nausea, vomiting and diarrhoea [41]. Table 1 presents comparative basic characteristics of GLP-1RAs. They differ in several other aspects, such as tolerability, efficacy in reducing weight and glycated haemoglobin (HbA1c) and immunogenicity [42].

**Table 1:** Characteristics of GLP-1RAs

Drug	Structure/homology To Human GLP-1	DPP-4 cleavage	Half-life	Recommendations in renal impairment	Antibodies
Exenatide [39, 43–45]	Substitution of alanine in position 2 by glycine 53% homology	Resistant	2–4 h (12 h for sustained release exenatide)	Not recommended in patients with GFR < 30 mL/min Sustained release exenatide is only licensed in mild-to-moderate renal impairment (GFR > 50 mL/min)	Anti-drug antibodies were more common, and titres were higher with exenatide once weekly than with exenatide twice daily
Lixisenatide [35, 46–50]	Exendin-4 elongated with a residue of 6 lysines attached to the C-terminus 50% homology	Resistant	2–3 h	Not recommended in patients with a GFR < 30 mL/min	56–60% of patients developed anti-drug antibodies, with no apparent effect on efficacy or safety
Liraglutide [51–56]	One amino acid substitution (Lys34Arg) with the addition of a C-16 acyl group (palmitoyl) attached to Lys26 via a glutamate linker 97% homology	Resistant	10–12 h	No restrictions or dose adjustments required	Low incidence of anti-drug antibodies
Albiglutide [57–59]	Composed of a GLP-1 (7–36) dimer fused to recombinant human albumin 95% homology	Resistant	5 days	No restrictions or dose adjustments required	Low incidence of anti-drug antibodies
Dulaglutide [42, 60, 61, 72]	Two DPP-4 resistant GLP-1 molecules covalently bound to a modified immunoglobulin 4 Fc fragment 90% homology	Resistant	5 days	No restrictions or dose adjustments required	Low incidence of anti-drug antibodies
Taspoglutide [64–66]	Alpha-aminoisobutyric acid substitution at positions 8 and 35 of the human GLP-1(7–36)NH2 that enhances enzymatic stability and potency 93% homology	Resistant	165 h	Renal impairment alters the pharmacokinetics of taspoglutide. The degree of renal impairment was associated with an increased exposure to taspoglutide and an increased risk of gastrointestinal adverse events	Incidence of anti-drug antibodies as high as 49%
Semaglutide [58–71, 73]	Acyl group with a steric diacid at Lys26 and a large synthetic spacer and modified by the presence of a alpha-aminobutyric acid in position 8 94% homology	Resistant	1 week	No restrictions or dose adjustments required for patients with renal impairment	Low incidence of anti-drug antibodies

Arg, arginine; DPP-4, dipeptidyl peptidase 4; GFR, glomerular filtration rate; GLP-1, glucagon-like peptide-1; Lys, lysine

### Exenatide

Exenatide was designed as a recombinant synthetic form of the peptide exendin-4, and in 2005, was the first GLP-1RA to be approved by the US Food and Drug Administration (FDA) for the treatment of T2DM [43]. Exenatide was first introduced as a twice-daily injection of 5 mcg for 1 month, followed by 5 mcg or 10 mcg. A new formulation that consisted of an extended-release, 2 mg once-weekly injection subsequently showed greater HbA1c reduction and better glucose control [39]. Exendin-4 crosses the blood–brain barrier more efficiently than native GLP-1 [44]. Following peripheral administration, exenatide has been shown to suppress food intake via vagal-dependent and vagal-independent pathways that result in the direct activation of GLP-1R in the central nervous system. Exenatide is primarily eliminated by the kidneys via renal filtration and enzymatic degradation in the tubules [45].

### Lixisenatide

Lixisenatide has the exedin-4 backbone and, as a result, an extended half-life. Approximately 55% of lixisenatide is bound to plasma proteins. Lixisenatide is administered as a 10 mcg once-daily injection, titrated to a dose of 20 mcg after 2 weeks [46]. In clinical trials, lixisenatide decreased HbA1c from baseline (by 0.7% to 0.94%), with an associated weight loss of 0.2–2.8 kg [35]. Lixisenatide has a major effect on postprandial glycaemia as a result of slowed gastric emptying. As monotherapy, it is effective in reducing HbA1c and fasting and postprandial glycaemia [47, 48]. Lixisenatide crosses the blood–brain barrier in animal studies following peripheral administration [49]. Elimination of lixisenatide is presumed to occur via renal filtration, tubular reabsorption and metabolic catabolism. No data are available about the nature and potential actions of its metabolites [50].

### Liraglutide

Liraglutide is a long-acting GLP-1 analogue, produced by recombinant DNA technology. It has 97% amino acid homology to endogenous human GLP-1. With a fatty-acyl moiety on the GLP-1 peptide backbone, it has an increased half-life as a result of DPP-4 resistance and non-covalent binding to serum albumin. Liraglutide should be initiated at a dose of 0.6 mg/day for 1 week and titrated on a weekly basis to a maximum of 1.8 mg/day. Liraglutide has been shown to significantly reduce fasting and postprandial glycaemia, as well as HbA1c levels. It is also associated with a low risk of hypoglycaemia and weight gain [51]. The significant weight loss reported in humans with and without T2DM has made liraglutide an attractive treatment option [52]. Liraglutide is not excreted via the kidneys, in contrast to GLP-1RAs based on exendin-4; consequently, its metabolism and excretion are mostly unaffected by a decreased glomerular filtration rate (GFR) [53, 54]. Liraglutide also crosses the blood–brain barrier and has anti-apoptotic, anti-inflammatory, antioxidant and neuroprotective effects, which may be of use in the treatment of neurodegenerative disease [49, 55]. GLP-1 [9–36] amide, a metabolite produced during liraglutide cleavage by DDP-4, mediates the anti-inflammatory effects of liraglutide observed after intracerebral haemorrhage [56].

### Albiglutide

Albiglutide is a long-acting GLP-1RA, developed by fusing a GLP-1 dimer to recombinant human albumin. A single substitution of alanine with glycine prevents albiglutide from being cleaved by DPP-4, which results in a longer half-life and allows for weekly administration [57]. Albiglutide has 97% homology to the amino acid sequence of GLP-1. It augments glucose-dependent insulin secretion and slows gastric emptying. The initial dose is 30 mg once weekly [58]. Albiglutide is a large biochemical entity that does not cross the blood–brain barrier, which may possibly underlie the fewer nausea events experience with albiglutide than with other GLP-1RAs [59]. However, albiglutide was voluntarily discontinued by its manufacturer for commercial reasons.

### Dulaglutide

Dulaglutide is a long-acting GLP-1RA with two identical GLP-1 analogue peptide chains linked to an immunoglobulin 4 heavy chain, thus limiting renal clearance. It is approximately 90% homologous to native human GLP-1 [60]. The alteration of the GLP-1 analogue results in a long half-life, as well as an improved solubility and reduced immunogenicity. The initial dose is 0.75 mg subcutaneously (SC) once weekly and may be increased to 1.5 mg once weekly. In a randomized placebo-controlled double-blind study conducted in 262 obese patients with diabetes, an HbA1c reduction of approximately 1.28–1.52% was observed, with a weight loss of 1.40–2.51 kg [61]. The larger molecular size of dulaglutide may hinder transport across the blood–brain barrier [42]. Cardiovascular benefits are still under investigation [62], but initial results have shown that dulaglutide does not increase the risk of major cardiovascular events in T2DM patients [63].

### Taspoglutide

Taspoglutide is a long-acting GLP-1RA considered to have potency equivalent to GLP-1 and is entirely resistant to DPP-4 degradation [64]. Taspoglutide was the first once-weekly GLP-1RA. Administration of taspoglutide 10 or 20 mg once weekly is associated with glycaemic control and weight loss. Data suggest that taspoglutide lowers HbA1c by approximately 1.1%. When compared with exenatide in the T-emerge 2 study, taspoglutide was associated with a higher incidence of gastrointestinal side effects and hypersensitivity reactions, which limited its use and clinical investigation [65, 66]. The manufacturer suspended late-stage trials in 2010 [67].

### Semaglutide

Semaglutide is a once-weekly GLP-1RA. The long half-life was achieved by applying the fatty acid acylation technology that provides specific high-affinity albumin binding, thus preventing renal filtration. Semaglutide was approved by the FDA for the treatment of T2DM in 0.5 mg and 1.0 mg doses. An oral form of semaglutide has also been tested with promising results [68–70]. It is associated with strong metabolic control and a decrease in body weight, with a safety profile characteristic of a GLP-1RA. In clinical trials, semaglutide reduced HbA1c by 1.5–1.8%, which was significantly more than active comparators, and was associated with a 4.5–6.4 kg weight loss [71].

## GLP-1 receptor stimulation: diabetes treatment and cardiovascular benefits

GLP-1RAs are indicated for glycaemic control in T2DM, are well tolerated and are subcutaneously administered. They mimic the effects of GLP-1 such as stimulation of insulin release and inhibition of glucagon release, both in a glucose-dependent manner. They also have other effects, including modulation of gastrointestinal motility and normalisation of fasting and postprandial insulin secretion. In most patients, GLP-1RAs promote weight loss over several months (1–4 kg on average) through satiety stimulation and reduction of caloric intake as a result of their direct effect on the reward and satiety areas in the central nervous system [74]. In summary, the key beneficial features of GLP-1RAs are weight loss and a relatively low risk of hypoglycaemia compared with other anti-hyperglycaemic agents [75–77].

Although small increases in heart rate are associated with the use of GLP-1RAs [78], GLP-1RAs may improve cardiovascular outcomes [79], in addition to anti-hyperglycaemic effect and promoting weight loss. Accumulating evidence in animals and humans shows that GLP-1R stimulation has beneficial effects on multiple organ systems in which GLP-1Rs exist, including the cardiovascular system [26]. Cytoprotection is among the pleiotropic actions described for GLP-1 in different cell types, including cardiomyocytes [80]. In isolated rodent hearts, stimulation of GLP-1R enhanced nitric oxide (NO) production, glucose uptake and coronary flow [29, 81]. Vasorelaxation was also well described in different rat vessels [82, 83]. These effects may provide some benefits during the acute phase of cardiac ischaemia.

GLP-1RAs may also have long-term benefits on the progression of the underlying atherosclerosis. For example, liraglutide treatment inhibited progression of early onset, low-burden atherosclerotic disease in the apolipoprotein E-deficient (ApoE^−/−^) mouse model [84]. Interesting results were reported by Rizzo and colleagues, with liraglutide significantly improving metabolic parameters (triglycerides) and carotid intima media thickness after 18 months in T2DM patients with metabolic syndrome [85]. In overweight patients with stable coronary artery disease and T2DM, liraglutide increased heart rate and reduced heart rate variability despite significant weight loss and improvement in metabolic parameters [86]. On the other hand, in a small sized and short-term trial, liraglutide did not improve the systolic function of the left ventricle during dobutamine stress echocardiography or the exercise capacity in patients with T2DM and stable coronary disease [87].

In heart failure models, GLP-1R stimulation has shown improvement in cardiac function [88, 89]. In patients with heart failure, 12 weeks of albiglutide improved oxygen consumption [90]. Moreover, administration of exenatide improved cardiac function in T2DM patients [91].

Another area of intense research is the neurotrophic and neuroprotective effects of GLP-1R stimulation in different animal models of stroke, with or without T2DM [92]. Li and colleagues showed that administration of exenatide reduced brain damage and improved functional outcome in a transient middle cerebral artery occlusion stroke in rodent models [93]. Exenatide was also shown to attenuate neuroinflammation and ameliorate warfarin-associated haemorrhagic transformation after cerebral ischaemia in mice [94]. Sato and colleagues demonstrated that, in rat models, liraglutide administered intraperitoneally 2.5 h after stroke onset induced neuroprotection through up-regulation of vascular endothelial growth factor (VEGF) and anti-oxidative effects [95]. Although this body of evidence is growing, it refers to functional outcomes. A number of concerns remain, including the exact mode of action of GLP-1RAs in the brain [96, 97], and more animal studies are needed to answer these questions.

While these results are very promising, large clinical studies are required to confirm that GLP-1RAs have clinically meaningful effects on cardiovascular outcomes and risk in patients with T2DM. In this review, we discuss the results of large clinical trials with exenatide, liraglutide, semaglutide and lixisenatide, in which the following exploratory cardiovascular outcomes were evaluated: cardiovascular death, nonfatal MI, nonfatal stroke, hospitalization for unstable angina or heart failure (3- or 4-point major adverse cardiovascular event [MACE]) [98].

## Reasons for improved cardiovascular outcomes

GLP-1RAs act on multiple organ systems in which GLP-1 receptors are present or not detected, including systems not involved in glucose regulation, such as the cardiovascular system. GLP-1RAs favourably modulate cardiovascular risk parameters, some of which are independent of weight loss, HbA1c reductions or the occurrence of severe hypoglycaemia. This potential advantage of GLP-1RAs has attracted attention, as intensive control of hyperglycaemia prevents microvascular complications, such as retinopathy, neuropathy and nephropathy, whereas macrovascular complications are most impacted by the control of traditional cardiovascular risk factors [99, 100]. Mechanisms that link hyperglycaemia and accelerated atherosclerotic disease have not been completely explained [101]. They seem to be mediated by vascular inflammation, endothelial dysfunction and oxidative stress [102]. However, hyperglycaemia alone may have only a mild contribution to the risk of cardiovascular events, as intensive glycaemic control showed a small effect on reducing non-fatal MI in up to 17% of the relative risk reduction [103, 104]. Therefore, optimal anti-hyperglycaemic treatment should include comprehensive control of multiple cardiovascular risk factors to improve macrovascular and microvascular complications, as well as the effects on reducing glycaemia. GLP-1RAs may mediate effects on cardiovascular outcomes through effects on other risk factors such as blood pressure, dyslipidaemia, platelet reactivity, endothelial dysfunction, and insulin sensitivity, as well as direct cardioprotective effects.

Clinical trial data show that GLP-1RAs can reduce blood pressure values: exenatide and liraglutide produce a mean decrease of 1–5 mmHg compared with placebo and other active comparators. These effects occur early after the start of treatment, suggesting that mechanisms other than weight loss may be involved [105–107].

Abnormal lipidaemia after a meal, referred to as postprandial dyslipidaemia, is linked to increased risk of morbidity and mortality as a result of CVD in individuals with or without T2DM [108]. Patients with T2DM present with abnormalities such as a higher peak and later decline of postprandial triglyceridaemia, features that have been associated with both early coronary artery and carotid artery atherosclerosis independently of traditional risk factors [109]. Insulin resistance in the liver and adipose cells, the compensatory hyperinsulinaemia, as well as hyperglycaemia and disturbed fatty acid metabolism, have been suggested as the main causes of postprandial dyslipidaemia [110]. In addition to enhancing insulin secretion, GLP-1RAs may reduce postprandial chylomicron overproduction in T2DM patients via mechanisms that include reduction in intestinal absorption of dietary lipids and enhanced hepatic fatty acid oxidation [111]. Exenatide and liraglutide have been reported to be equally effective in lowering postprandial dyslipidaemia, an effect observed immediately after initial administration [112]. In a double-blind, randomized, placebo-controlled, crossover study with 35 subjects who exhibited impaired glucose tolerance (n = 20) or had recent-onset T2DM (n = 15), a single subcutaneous injection of exenatide strongly and consistently inhibited the postprandial increase of proatherogenic lipids and lipoproteins [113]. It is possible that the effect of GLP-1RAs on postprandial dyslipidaemia may contribute to the attenuation of macrovascular risk, as well as its effects on body weight, blood pressure and glycaemia. From an academic point of view, it is relevant to discern the specific contribution of this effect, whereas in the clinical field, the possibility of acting on a wide range of risk factors that contribute to insulin resistance is one of the most important characteristics of this drug class.

In the context of T2DM, disturbances in platelet aggregation may be caused by decreased NO bioavailability and endothelial insulin resistance, as well as the presence of the NO signalling cascade within the platelet, contributing to platelet hyperactivity [120]. In the presence of oxidative stress, platelet hyperactivity has a major effect on the risk of atherothrombotic events. In a study in cultured human megakaryocytes, exenatide increased the release of cyclic AMP and further inhibited platelet aggregation induced by thrombin, adenosine diphosphate or collagen [114–117]. Translationally relevant, these findings provide novel insights regarding the ability of GLP-1RAs to attenuate platelet aggregation and thrombosis by the activation of endothelial NO synthase and NO production. It is difficult to estimate the contribution of the antiplatelet effect of GLP-1RAs in reducing the risk of MACE when most patients included in the trials were on antiplatelet therapy. This effect would potentially acquire greater relevance for individuals in primary prevention settings, such as 20% of the LEADER (Liraglutide Effect and Action in Diabetes: Evaluation of Cardiovascular Outcome Results—A Long Term Evaluation) trial participants.

In T2DM patients, severe hypoglycaemia is considered a strong predictor of macrovascular events and death. During hypoglycaemia, cellular glucose deprivation or activation of the sympathoadrenal response may lead to arrhythmias, inflammation, or endothelial dysfunction and may favour a prothrombotic state [117]. Insulin resistance increases the risk of cardiovascular disease via a spectrum of mechanisms that include fatty acid efflux, inflammation, endothelial dysfunction and atherogenic dyslipidaemia [118]. GLP-1RAs improve glucose homeostasis mainly through their most well-characterised effect, augmentation of glucose-stimulated insulin secretion, but also due to increase in insulin sensitivity and in the risk of hypoglycaemia. In beta cells, GLP-1 stimulates insulin secretion, insulin gene transcription, islet cell growth and neogenesis [119].

The cardiovascular effects of GLP-1RAs may also be mediated by effects on endothelial dysfunction. This early abnormality in atherosclerotic disease may precede T2DM diagnosis by many years [120]. Although the hallmark of endothelial dysfunction is reduction in NO bioavailability, the functional decline of endothelial cells reaches a much broader scope, influencing for example, thrombogenicity and inflammation [121]. GLP-1RA treatment may act directly on endothelial cells, thereby improving elements of endothelial function via direct and indirect mechanisms. In 2004, Nyström and colleagues showed for the first time via a flow-mediated dilation (FMD) technique, that GLP-1 infusion ameliorates endothelial dysfunction in T2DM patients with established coronary artery disease [122]. Exenatide elicits NO production in endothelial cells through activation of GLP-1R and AMP-activated protein kinase (AMPK) and, thus, induces vasorelaxation even when levels of blood glucose or lipid levels are high [123]. Similarly, in vivo studies have demonstrated improvement in endothelial cell function in mice treated with liraglutide, as estimated by an increased endothelial NO synthase expression and a decreased production of ICAM-1 (intercellular adhesion molecules-1), a GLP-1R-dependent effect [124]. Moreover, liraglutide attenuated the induction of PAI-1 (plasminogen activator inhibitor type-1) and VCAM (vascular adhesion molecule) expression in human vascular endothelial cells in vitro [132]. Clinical studies with underpowered sample sizes have shown mixed results. In subjects with T2DM (n = 20), exenatide, compared with glimepiride, improved brachial artery function after a 4-month treatment evaluated with FMD [125]. Despite significant improvements in body composition and glycaemic control, treatment with exenatide or liraglutide for obese T2DM patients (n = 11) did not improve vascular function parameters after 6 months [126]. Certainly, the effect of GLP-1RA on endothelial function in humans cannot be estimated by these studies because of the low statistical power or the limitations that led to the improvement of the FMD technique [127]. The relevance for this type of studies must be considered from the viewpoint of the feasibility of FMD as a surrogate outcome and, even more so, by the mechanistic ideas that their findings may reveal.

In the setting of ischaemia–reperfusion (IR) injury, exenatide has been shown to protect IR-induced endothelial dysfunction (measured by FMD) through the opening of adenosine triphosphate-sensitive potassium channels in a human IR injury model [128]. Moreover, administration of exenatide at the time of reperfusion in patients with ST-segment elevation myocardial infarction (STEMI) treated with primary percutaneous coronary intervention increases myocardial salvage, thus showing a cardioprotective effect [129]. Similar results were seen with liraglutide [130]. In 92 patients, a short course of liraglutide in STEMI patients treated with primary percutaneous coronary intervention was associated with mild improvement in the left ventricular ejection fraction and favourable changes in markers of inflammation and endothelial function [131].

At least part of this effect occurs via GLP-1R and is mediated by the AMPK/phosphoinositide 3-kinase (PI3K)-protein kinase B (Akt) pathway [132]. Accordingly, endothelial and myocardial prevention of IR lesions has been observed with exendin-4, GLP-1 and all tested GLP-1RAs. Nevertheless, myocardial protection for IR injury is equally observed in GLP-1R knock-out animal models [29, 133], which suggests that metabolites and indirect pathways equally contribute to this outcome. Moreover, GLP-1 [9–36] amide and exendin-4 demonstrated myocardial protection that was only partially inhibited by GLP-1R blockade [134].

Accumulating evidence suggests that the actions of GLP-1 degradation products are mediated through novel receptors distinct from traditional GLP-1R or passive transport through cellular membranes. GLP-1[9–36]amide, for example, may interact directly with the CD36/fatty acid translocator [30]. In addition, because of its amphipathic structure (N-terminal domain is hydrophobic and the C-terminal is positively charged), the small metabolite GLP-1[28–36]amide can cross cell membranes and, via energy-independent mechanisms, may interact directly with intracellular organelles such as mitochondria [135]. The triggering of the mitochondrial response by GLP-1[28–36]amide results from the binding between its C-terminal domain and the consensus mitochondrial targeting sequence or between the tryptophan residue at position 31 of the peptide and proteins present in the mitochondria [30, 136]. In summary, both direct and indirect actions of GLP-1 on mitochondria favour anti-apoptotic and anti-oxidant actions, in addition to modifying fatty acid oxidation and energy expenditure [30]. The protective effect of GLP-1RAs on myocardial tissue that does not express the GLP-1R classical pathway suggests that distinct receptors and potentially metabolites of these agonists may contribute to their beneficial effects on clinical outcomes.

## Heterogeneity and impact of cardiovascular safety studies

In 2008, the FDA published diabetes guidelines for the pharmaceutical industry, thus setting new expectations for the development of drugs for T2DM. Due to safety concerns, the guidance mandates that all new antidiabetic drugs rule out excess cardiovascular risk in long-term cardiovascular outcome trials (CVOTs). The requirements for CVOTs include, among others, selection of patients from high-risk populations, including individuals with established cardiovascular disease, multiple risk factors and renal impairment. Trials must include at least 2 years of cardiovascular safety data [137].

Since 2008, the results of five large-scale randomized trials that assessed major cardiovascular outcomes have been reported with a number of GLP-1RAs: exenatide (EXSCEL: EXenatide Study of Cardiovascular Event Lowering), lixisenatide (ELIXA: Evaluation of Lixisenatide in Acute Coronary Syndrome), liraglutide (LEADER), semaglutide (SUSTAIN-6: Trial to Evaluate Cardiovascular and Other Long-term Outcomes with Semaglutide in Subjects with Type 2 Diabetes) and, more recently, albiglutide (HARMONY Outcomes: Albiglutide and cardiovascular outcomes in patients with T2DM and cardiovascular disease) and dulaglutide (REWIND: Researching cardiovascular Events with a Weekly INcretin in Diabetes). These trials were powered to assess non-inferiority or adequately detect differences between the drug and placebo regarding cardiovascular outcomes in patients with T2DM at high risk for cardiovascular events or established CVD. Thus, they fundamentally differed from most of the studies included in the pooled analyses and meta-analyses of incretins, which tended to include patients at low risk for cardiovascular events. Table 2 provides a summary of the main results.

**Table 2:** Characteristics of GLP-1RA trials [100, 135–138]

	EXSCEL	ELIXA	LEADER	SUSTAIN-6	HARMONY	REWIND
GLP-1RA	Exenatide	Lixisenatide	Liraglutide	Semaglutide (SC)	Albiglutide	Dulaglutide
N	14,752	6068	9340	3297	9463	9901
Inclusion criteria	T2DM With prior cardiovascular events and/or with or without known cardiovascular risk factors	T2DM Acute coronary event within 180 days prior to randomization	T2DM Previous CVD or CKD High risk CVD	T2DM Previous CVD or CKD High-risk CVD	T2DM Previous CVD High-risk CVD	T2DM Previous CVD High-risk CVD
Study design	Phase 3/4 multicentre, randomised, double-blind, placebo-controlled, parallel-group; non-inferiority, superiority (hierarchical analysis)	Multicentre, randomised, double-blind, placebo-controlled; non-inferiority, superiority	Multicentre, double-blind, placebo-controlled; non-inferiority, superiority (hierarchical analysis)	Multicentre, double-blind, placebo-controlled; non-inferiority, superiority testing was not part of the pre-specified analysis	Multicentre, randomised, double-blind, placebo-controlled; non-inferiority, superiority testing was pre-specified	Multicentre, randomised, double-blind, placebo-controlled; non-inferiority, superiority testing was pre-specified
Primary outcome	3-point MACE: cardiovascular death, non-fatal MI, non-fatal stroke	4-point MACE: cardiovascular death, non-fatal MI, non-fatal stroke, hospitalisation for unstable angina	3-point MACE: cardiovascular death, non-fatal MI, non-fatal stroke	3-point MACE: cardiovascular death, non-fatal MI, non-fatal stroke	3-point MACE: cardiovascular death, non-fatal MI, non-fatal stroke	3-point MACE: cardiovascular death, non-fatal MI, non-fatal stroke
Results	Exenatide group: 11.4% Placebo group: 12.2% HR 0.91; 95% CI 0.83–1.00 P < 0.001 for non-inferiority, P = 0.06 for superiority	Lixisenatide group: 13.4% Placebo group: 13.2% HR 1.02; 95% CI 0.89–1.17 P < 0.001 for non-inferiority, P = 0.81 for superiority	Liraglutide group: 13.0% Placebo group: 14.9% HR 0.87; 95% CI 0.78–0.97 P < 0.001 for non-inferiority, P = 0.01 for superiority	Semaglutide group: 6.6% Placebo group: 8.9% HR 0.74; 95% CI 0.58–0.95 P < 0.001 for non-inferiority, P = 0.02 for superiority	Albiglutide group: 7.1% Placebo group: 9.0% HR 0.78; 95% CI 0.68–0.90 P < 0.001 for non-inferiority, P = 0.0006 for superiority	Preliminary results reported as a positive trial
Additional findings			Reduction in all-cause mortality in the liraglutide group (8.2% vs. 9.6% in placebo group; HR 0.85; 95% CI 0.74–0.97; P = 0.02) Reduction in CV death in the liraglutide group (4.7% vs. 6.0% in the placebo group; HR 0.78; 95% CI 0.66–0.93; P = 0.007)	Reduction in nonfatal stroke: Semaglutide group: 1.6% Placebo group: 2.7% HR 0.61; 95% CI, 0.38–0.99 P = 0.04		

CI, confidence interval; CKD, chronic kidney disease; CVD, cardiovascular disease; GLP-1RA, glucagon-like peptide-1 receptor agonist; HR, hazard ratio; MACE, major adverse cardiovascular event; SC, subcutaneous; T2DM, type 2 diabetes mellitus

## EXSCEL

Before the EXSCEL trial was completed, the exploratory results from a large uncontrolled study population of 39,275 patients suggested that patients treated with exenatide twice daily had a significantly lower rate of cardiovascular events and hospitalisations than patients treated with other glucose-lowering medications [139]. In contrast to these preliminary findings, the results from the subsequent randomised, placebo-controlled EXSCEL trial failed to demonstrate a cardiovascular advantage. The EXSCEL trial enrolled the largest and most inclusive patient population of any cardiovascular outcome trial of the GLP-1RA class. In T2DM patients with an elevated cardiovascular risk, exenatide 2 mg weekly was compared with placebo. EXSCEL was unique in the inclusion of patients with a broad range of cardiovascular risks, including 26.9% without known cardiovascular events. Fewer cardiovascular events occurred in the exenatide group, thus showing cardiovascular safety; however, exenatide failed to show a significant cardiovascular benefit, with the overall results not significantly different from the placebo group. No significant differences were identified in severe hypoglycaemia, pancreatitis, pancreatic cancer or medullary thyroid cancer between treatment and placebo [140].

### ELIXA

The ELIXA trial included subjects randomised to receive lixisenatide (starting dose 10 mcg for 2 weeks, maximum dose 20 mcg) or placebo who were followed for a median of 25 months. The outcome of this study was the time to first MACE using a composite of 4 cardiovascular outcomes, and the study was powered to detect both non-inferiority and superiority. ELIXA was the first randomised, double-blind, non-inferiority cardiovascular outcome trial of a GLP-1RA to be reported. Only patients with previous acute coronary events were included in the trial. The results indicated similar rates of the primary composite outcome in both groups and no significant differences between the groups when components of the primary outcome were assessed independently. Moreover, there were no significant differences between the groups regarding hospitalisations for heart failure; this pattern persisted when analysed with the inclusion of hospitalisations for coronary revascularisation. Lixisenatide was not associated with an increased rate of serious adverse events or severe hypoglycaemia and exhibited a modest benefit on weight control [141].

### LEADER

The LEADER trial investigated cardiovascular outcomes in patients with T2DM treated with liraglutide or placebo in addition to standard care [137]. Patients were randomised to receive either liraglutide 1.8 mg (or maximum tolerated dose) or placebo once daily. The median follow-up for each group was 3.8 years. Approximately 80% of patients in each arm had established CVD or CKD and 20% had no established CVD disease but cardiovascular risk factors. The primary outcome was the time to occurrence of a MACE, using a composite of three cardiovascular outcomes. In patients who received liraglutide, there was a reduction in both all-cause mortality and 3-point MACE. Although these patients had a lower overall risk of death from cardiovascular causes, the rates of non-fatal MI (6.0% vs. placebo 6.8%; hazard ratio [HR] 0.88; 95% confidence interval [CI] 0.7–1.03; P = 0.11) and non-fatal stroke (3.4% vs. placebo 3.8%; HR 0.89; 95% CI 0.72–1.11; P = 0.30) remained similar. In patients with established CVD, the primary endpoint rates were 14% in the liraglutide and 16.7% in the placebo group, which corresponds to an absolute risk reduction of 2.7% or an number needed to treat (NNT) of 37. There was also no difference between the groups in the rates of heart failure hospitalisations. The incidence of a composite outcome of renal or retinal microvascular events was lower in the liraglutide group, driven by a lower rate of nephropathy events in this group than in the placebo group (1.5 vs. 1.9 events per 100 patient-years, respectively; HR 0.78; 95% CI, 0.67–0.92; P = 0.003) [138].

Kaplan–Meier cumulative event curves for 3-point MACE started to separate lately at 12–18 months from randomisation. The same was true for death from cardiovascular causes and death from any cause, which may suggest that at least part of liraglutide effect on cardiovascular events was mediated by its anti-atherosclerotic effect. However, in comparison to classic diabetes studies (aimed at achieving near-normoglycaemia) in patients with T2DM, the cardiovascular effects of GLP-1a were detected relatively early [138]. In the United Kingdom Prospective Diabetes Study (UKPDS), intensive glucose-lowering therapy in patients with T2DM was associated with a reduced risk of clinically evident microvascular complications [103]. A post-trial extended follow-up suggested that a significant reduction in macrovascular events may occur at long-term [13]. The ACCORD (Action to Control Cardiovascular Risk in Diabetes) study was a 20-year investigation comparing the effects of diet to sulfonylureas or insulin [142]. The pre-specified primary outcome was the first occurrence of non-fatal MI, non-fatal stroke or death from cardiovascular causes. Differences between the primary outcome curves and the curves for death from any cause took years to emerge. Intensive glucose-lowering was associated with a reduction in the primary combined outcome but this was also associated with a significant increase in mortality. The ADVANCE (Action in Diabetes and Vascular Disease: Preterax and Diamicron Modified Release Controlled Evaluation) study also compared intensive to standard therapy in T2DM. The primary outcomes included a composite of macrovascular events (non-fatal MI, non-fatal stroke or cardiovascular-related death) and major microvascular events (new or worsening nephropathy or retinopathy). Although there was a reduction in the microvascular outcome, there was no change in the incidence of macrovascular events after 5 years [104]. The Veterans Affairs Diabetes Trial (VADT) showed that intensive glucose lowering, as compared with standard therapy, did not significantly reduce the rate of major cardiovascular events among 1791 military veterans. After nearly 10 years of post-trial extended follow-up, patients with T2DM who had been randomly assigned to intensive glucose control had fewer major cardiovascular events, although no improvement was seen in the rate of overall survival [143].

The beneficial effects of liraglutide on cardiovascular events and death appear to be independent of the lower incidence of severe hypoglycaemia, reported in a post hoc analysis of the LEADER study [144].

### SUSTAIN-6

The SUSTAIN-6 randomised, placebo-controlled trial was designed to assess the non-inferiority of semaglutide compared with placebo for cardiovascular safety in patients with T2DM. Baseline CVD was present in 83% of patients. The SUSTAIN-6 trial demonstrated that treatment with once-weekly semaglutide 0.5 or 1.0 mg subcutaneously for 2 years significantly reduced cardiovascular risk. Although the study was not designed to test superiority, the semaglutide group had significantly lower rates of cardiovascular events than placebo. This reduction in MACE was driven by significantly more non-fatal stroke in the placebo group than in the semaglutide group, whereas there were no differences in death from a cardiovascular cause or in non-fatal MI. The rates of nephropathy were lower with semaglutide: new or worsening nephropathy was 3.8% in the semaglutide group and 6.1% in the placebo group (HR 0.64; 95% CI, 0.46–0.88; P = 0.005). Moreover, complications of retinopathy were unexpectedly higher with semaglutide (3.0% vs. 1.8% in the placebo group; HR 1.76; 95% CI, 1.11–2.78; P = 0.02). Subgroup analyses showed that retinopathy complications were more likely in patients who had rapid HbA1c lowering during the trial regardless of the treatment arm [145].

### HARMONY

The HARMONY study was the latest large CVOT trial published. It enrolled 9463 patients to receive either albiglutide, administered subcutaneously once weekly or placebo in a 1:1 ratio over a median period of 1.6 years [146]. Included patients had T2DM, were 40 years old or older and had a diagnosis of established disease of the coronary, cerebrovascular or peripheral arterial circulation. Patients with severe CKD were excluded. Albiglutide reduced the risk of MACE by 22% (95% CI 10–32%) when added to standard care in patients with T2DM and CVD. The HRs were 0.93 (95% CI 0.73–1.19) for death from cardiovascular causes, 0.75 (0.61–0.90) for myocardial infarction and 0.86 (0.66–1.14) for stroke. Tolerability and safety were acceptable. Despite the short duration of follow-up, this study added more evidence to support the hypothesis that GLP-1-receptor agonists can improve cardiovascular outcomes in patients with T2DM.

**Fig. 1:**
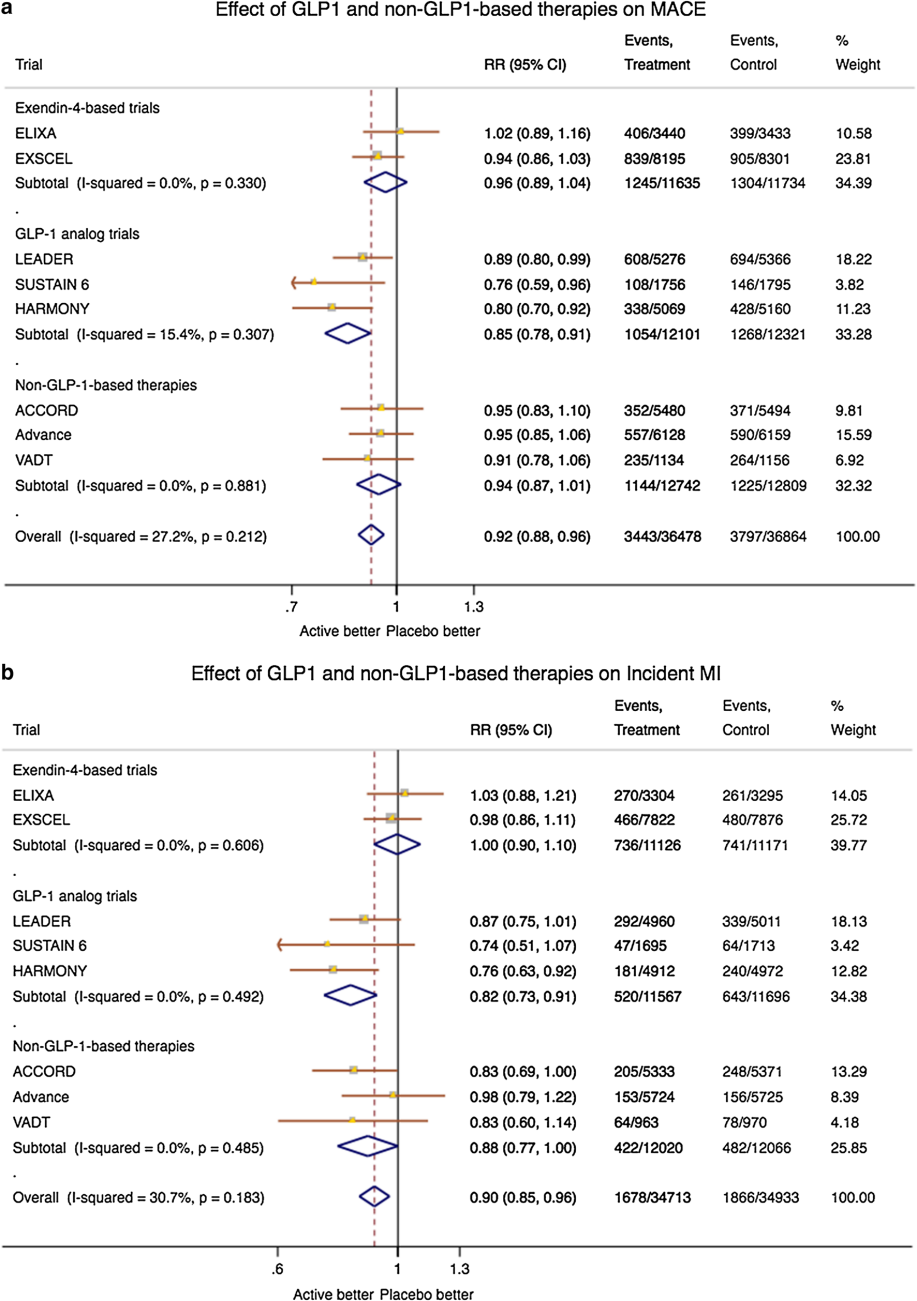
Forest plots showing the effects of GLP-1- and non-GLP-1-based therapies on **a** MACE, **b** myocardial infarction

**Fig. 2:**
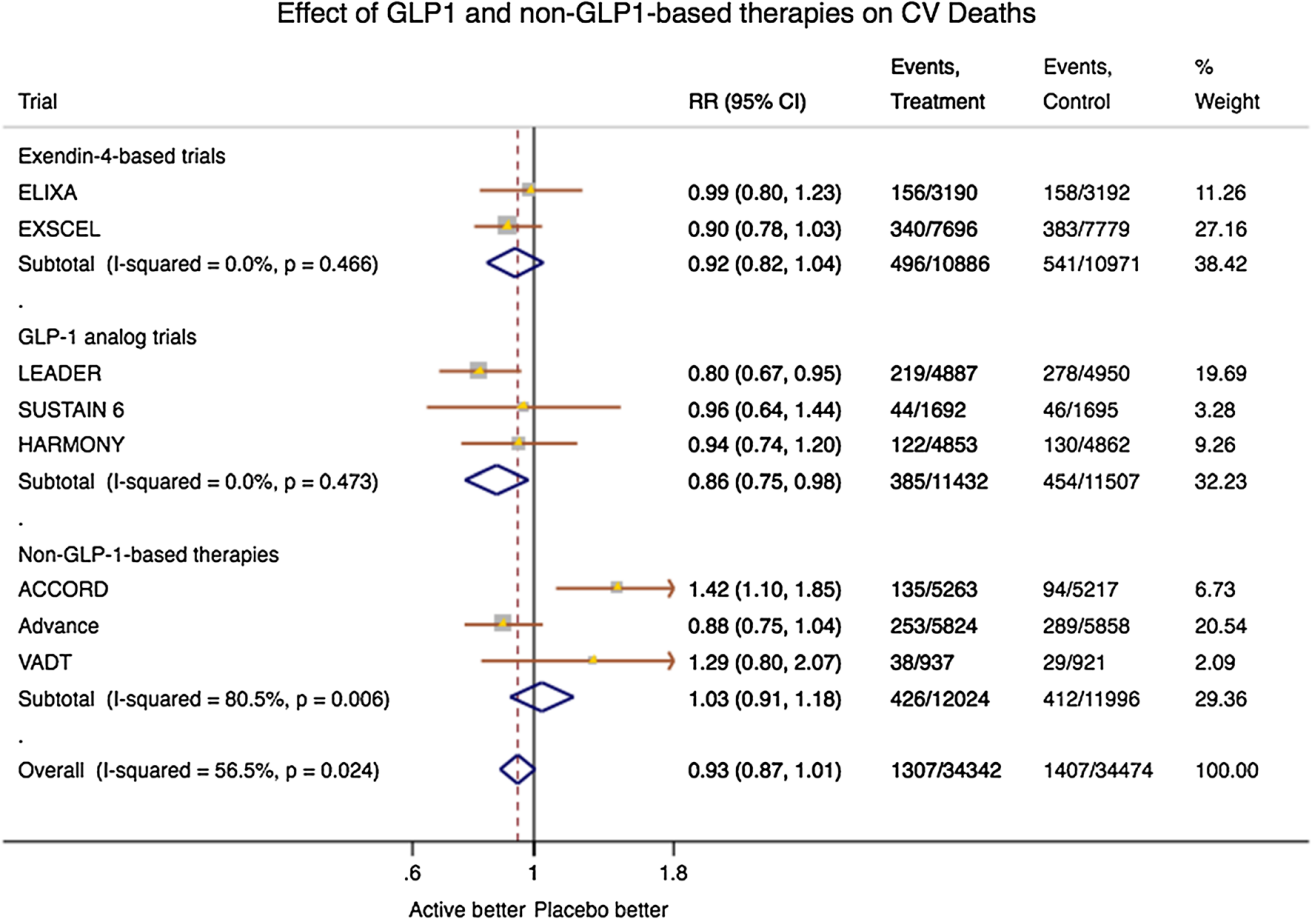
Forest plots showing the effects of GLP-1- and non-GLP-1-based therapies on cardiovascular death

The cardiovascular outcomes associated with exedin-4, GLP-1- and non-GLP-1-based therapies are depicted in the forest plot in Figs. 1 and 2. Compared with exedin-4-based therapies and non-GLP1-based therapies, the combined results from LEADER, SUSTAIN-6 and HARMONY studies showed a significantly reduced risk of MACE, MI and cardiovascular death, supporting the possible beneficial cardiovascular effect of GLP-1-based therapies. Heterogeneity among trials for the analysed end points was not significant, except for cardiovascular death that became significantly lower only after GLP-1-based therapies. Although this finding is consistent with the more pronounced effect of GLP-1 based therapies on the incidence of MACE and MI, one cannot rule out that the differences are simply the result of variations in the populations studied.

### REWIND

The REWIND trial was designed to evaluate the effect of the addition of once-weekly administered dulaglutide on the standard of care for patients with T2DM in the incidence of cardiovascular events [147]. Patients with HbA1c ≤ 9.5% using a maximum of 2 classes of antidiabetic drugs, aged 50 years or older if they had previous cardiovascular disease or 60 years or older if they had at least 2 other cardiovascular risk factors were enrolled. Individuals with an estimated glomerular filtration rate (eGFR) < 15 mL/min/1.73 m^2^, a gastric emptying anomaly, anterior pancreatitis, liver disease, or medullary carcinoma of the thyroid gland were excluded. The primary cardiovascular endpoint of the REWIND study was 3-P MACE, i.e. cardiovascular death or non-fatal MI or nonfatal stroke. Secondary endpoints were each component of the primary composite endpoint, retinal or renal disease, hospitalization for unstable angina, heart failure requiring hospitalization, and all-cause mortality. Safety outcomes included acute pancreatitis, severe gastrointestinal pain, pancreatic, thyroid, or other cancer, severe hypoglycaemia. The study was completed and its positive result in reducing the primary combined event was preliminarily released. Details of your results should be published later in this year.

## Critical overview of the RCTs

Although these trials have been conducted separately, with diverse types of patient cohorts and different degrees of background CVD, the patient populations in all trials were more similar than different. LEADER, SUSTAIN-6 and HARMONY reported favourable outcomes. The differences in cardiovascular outcomes were apparent by 12 months, which suggests that the effects observed were likely unrelated to differences in the glucose-lowering efficacy. The heterogeneity in cardiovascular outcomes may be the result of differences in the study designs and populations, treatment durations, pharmacokinetic and pharmacodynamic properties or structural similarity to human GLP-1 [148]. In these large clinical trials of GLP-1RAs, reduced stroke incidence was only seen with semaglutide in the SUSTAIN-6 study [149], although stroke incidence was included in the efficacy outcomes, as MACE, in all such trials.

The ELIXA study had a shorter duration, and because lixisenatide is short-acting, patients were not exposed to the drug for most of the day. However, it must be considered that ELIXA patients had recently manifested acute coronary syndrome (ACS; < 180 days) and, as such, had a significant driver of cardiovascular risk compared with stable chronic patients. After an ACS event, the homing of monocytes and activation of the systemic inflammatory response acutely intensify the vulnerable phenotype of coronary atherosclerotic plaques that causes approximately 50% of recurrent events in the first year of follow-up. In addition, the persistent increase in thrombogenicity favours the recurrence of coronary events. If the benefit in the protection from ischemia reperfusion injury is most clearly expressed among individuals at high risk for ACS, non-ischaemic effects of these GLP-1RAs may be camouflaged by the decreased signal-to-noise ratio.

As previously discussed, the EXSCEL trial included a wide range of patients with T2DM, and the primary endpoint did not reach statistical significance (P = 0.06). The secondary analysis of total mortality showed a reduction of 14% with alpha < 0.05; however, it was not considered significant by a well-established hierarchical criterion in which analyses of the secondary endpoints can only be made when the primary outcome is significant. Post hoc exploratory analyses must always be considered with caution. Without disregarding this fact, it remains noteworthy that the primary outcome reached statistical significance with approximately 10% relative risk reduction following the exclusion of 30% of patients who were on primary prevention. Similarly, statistically significant heterogeneity between primary and secondary prevention patients was identified in the LEADER trial, which suggests a patient-related effect rather than a drug-related mechanism. More research is required to confirm whether the cardiovascular benefits persist in T2DM patients without established CVD, as well as in patients without T2DM.

Concerning heart failure, to date this issue has been assessed as a secondary safety objective in large clinical trials of patients with T2DM. In contrast to the results obtained in many animal models, results in humans are not remarkable [80]. However, there was no increase in the risk of hospitalisation due to heart failure in all the prospective cardiovascular outcome trials discussed above, indicating a neutral effect. The FIGHT trial was a small study enrolling 300 HF patients with reduced ejection fraction (≤ 40%) and recent hospitalization to receive either liraglutide or placebo daily. The use of liraglutide did not change the incidence of the combined primary endpoint death rate or rate of hospitalization [150]. Several studies are being conducted to evaluate the effect of GLP-1a on cardiac function. The ongoing clinical trial Effects of Liraglutide in Young Adults with Type 2 Diabetes (LYDIA) is investigating changes in cardiac structure and function in younger (ages 18–40) obese patients with T2DM treated with liraglutide to determine effects on cardiac diastolic function [151].

## Conclusion and future perspectives

The use of GLP-1RAs is now a well-established practice in both the early and late stages of T2DM. However, further research into several significant aspects is on-going, such as improvement of treatment adherence with oral and inhaled formulations. A trial investigating the cardiovascular safety of oral semaglutide in patients with T2DM (NCT02692716) is currently on-going [152]. Other interesting areas of research include the use of osmotic pump systems, the combination of GLP-1RAs with basal insulin therapy and the potential use of GLP-1RAs in the treatment of type 1 diabetes. Sodium-glucose co-transporter 2 inhibitors (SGLT2i), like GLP-1RAs, have been shown to reduce cardiovascular mortality and hospitalisation for heart failure in high-risk patients with T2DM [153]. A recent network meta-analysis showed that the small difference in the decrease in cardiovascular mortality between SGLT2i and GLP-1RAs was not statistically significant [154]. Although provocative, this type of meta-analysis is intrinsically tied to biases related to clinical severity and differences in complementary treatments, particularly in the placebo groups. For example, the incidence of MACE in the placebo group of the SGLT2i studies is higher than that of the GLP-1a studies, generating substantial, non-statistically scalable heterogeneity. The combination of GLP-1RAs and SGLT2i is an exciting speculation for the future, as this combined therapy may produce additive cardiovascular benefits in patients with T2DM [155]. The development of other peptide hormones that stimulate insulin secretion and regulate appetite is promising, such as dual co-agonists developed in a single molecule, the stimulation of both the GLP-1 and peptide-YY receptor pathways and the co-administration of glucagon and GLP-1 [42].

Emerging data on the cardiovascular benefits associated with GLP-1RA treatment in patients with T2DM and an elevated risk of CVD are encouraging, particularly for liraglutide and semaglutide. However, the definition of cardiovascular risk or CVD varies across the trials, as do baseline characteristics, trial duration, routine care and event rates. Furthermore, the associated diseases and disease severity differ among the enrolled patients, which makes comparison of cardiovascular outcomes difficult. GLP-1RAs have been shown to have advantageous pleiotropic effects, acting in multiple organ systems, with potential cardiovascular benefits. Large trials are still necessary to confirm the potential efficacy of GLP-1RAs in improving the stroke outcomes specifically [95]. In addition, while stroke prevention should be the goal in a high-risk population such as T2DM patients, it is also extremely important to know if treatment is able to decrease damage and improve functional outcomes after stroke. To this end, exenatide is being evaluated in a study of hyperglycemic patients with suspected stroke and in patients receiving thrombolytic therapy for stroke [156].

GLP-1RAs have transformed the way we view diabetes treatment. Although the published data are very promising, important aspects require elucidation, including the specific mechanisms by which these drugs may generate cardiovascular benefits. The structural differences between the various GLP-1RAs may result in unique clinical profiles and should be considered when selecting a GLP-1RA for an individual patient. This new patient-oriented approach may benefit T2DM patients with high cardiovascular risk, especially for safety reasons.

## Abbreviations

ACCORD: Action to Control Cardiovascular Risk in Diabetes
ACS: acute coronary syndrome
ADVANCE: Action in Diabetes and Vascular Disease: Preterax and Diamicron Modified Release Controlled Evaluation
AMP: adenosine monophosphate
AMPK: AMP-activated protein kinase
CI: confidence interval
CKD: chronic kidney disease
CVD: cardiovascular disease
CVOT: cardiovascular outcome trial
DPP-4: dipeptidyl peptidase 4
DEVOTE: trial comparing cardiovascular safety of insulin degludec vs. insulin glargine in patients with type 2 diabetes at high risk of cardiovascular events
ELIXA: evaluation of lixisenatide in acute coronary syndrome
EXSCEL: exenatide study of cardiovascular event lowering
FDA: US Food and Drug Administration
FMD: flow-mediated dilation
GFR: glomerular filtration rate
GIP: glucose-dependent insulinotropic polypeptide
GLP-1: glucagon-like peptide-1
GLP-1R: GLP-1 receptor
HbA1c: glycated haemoglobin
HR: hazard ratio
IR: ischaemia-reperfusion
LEADER: liraglutide effect and action in diabetes: evaluation of cardiovascular outcome results
MACE: major adverse cardiovascular event
MI: myocardial infarction
NO: nitric oxide
ORIGIN: outcome reduction with initial glargine intervention
RA: receptor agonist
RCT: randomized clinical trial
STEMI: ST-segment elevation myocardial infarction
SUSTAIN-6: trial to evaluate cardiovascular and other long-term outcomes with semaglutide in subjects with type 2 diabetes
T2DM: type 2 diabetes
VADT: Veterans Affairs Diabetes Trial
UKPDS: United Kingdom Prospective Diabetes Study

## References

[1] IDF Diabetes Atlas 8th edition. 2017. https://www.idf.org/e-library/welcome.html. Accessed 16 Feb 2018.

[2] Wild S, Roglic G, Green A, Sicree R, King H (2004). Global prevalence of diabetes: estimates for the year 2000 and projections for 2030. Diabetes Care.

[3] Kahn SE, Cooper ME, Del Prato S (2014). Pathophysiology and treatment of type 2 diabetes: perspectives on the past, present, and future. Lancet.

[4] Defronzo RA (2009). Banting Lecture. From the triumvirate to the ominous octet: a new paradigm for the treatment of type 2 diabetes mellitus. Diabetes.

[5] Paneni F, Beckman JA, Creager MA, Cosentino F (2013). Diabetes and vascular disease: pathophysiology, clinical consequences, and medical therapy: part I. Eur Heart J.

[6] Naudi A, Jove M, Ayala V, Cassanye A, Serrano J, Gonzalo H (2012). Cellular dysfunction in diabetes as maladaptive response to mitochondrial oxidative stress. Exp Diabetes Res..

[7] Wei M, Gaskill SP, Haffner SM, Stern MP (1998). Effects of diabetes and level of glycemia on all-cause and cardiovascular mortality. The San Antonio Heart Study. Diabetes Care..

[8] Rutter MK, Nesto RW (2011). Blood pressure, lipids and glucose in type 2 diabetes: how low should we go? Re-discovering personalized care. Eur Heart J..

[9] Sarwar N, Gao P, Seshasai SRK, Gobin R, Kaptoge S, The Emerging Risk Factors Collaboration (2010). Diabetes mellitus, fasting blood glucose concentration, and risk of vascular disease: a collaborative meta-analysis of 102 prospective studies. Lancet..

[10] Rydén L, Grant PJ, Anker SD, Berne C, Cosentino F, Authors/Task Force Members (2013). ESC Guidelines on diabetes, pre-diabetes, and cardiovascular diseases developed in collaboration with the EASD: the Task Force on diabetes, pre-diabetes, and cardiovascular diseases of the European Society of Cardiology (ESC) and developed in collaboration with the European Association for the Study of Diabetes (EASD). Eur Heart J..

[11] Gu K, Cowie CC, Harris MI (1999). Diabetes and decline in heart disease mortality in US adults. JAMA.

[12] Juutilainen A, Lehto S, Rönnemaa T, Pyörälä K, Laakso M (2005). Type 2 diabetes as a “coronary heart disease equivalent”: an 18-year prospective population-based study in finnish subjects. Diabetes Care.

[13] Holman RR, Paul SK, Bethel MA, Matthews DR, Neil HAW (2008). 10-year follow-up of intensive glucose control in type 2 diabetes. N Engl J Med.

[14] Holman RR, Paul SK, Bethel MA, Neil HA, Matthews DR (2008). Long-term follow-up after tight control of blood pressure in type 2 diabetes. N Engl J Med..

[15] Colhoun HM, Betteridge DJ, Durrington PN, Hitman GA, Neil HA, Livingstone SJ (2004). Primary prevention of cardiovascular disease with atorvastatin in type 2 diabetes in the Collaborative Atorvastatin Diabetes Study (CARDS): multicentre randomised placebo-controlled trial. Lancet..

[16] Rawshani A, Rawshani A, Franzén S, Eliasson B, Svensson A-M, Miftaraj M (2017). Mortality and cardiovascular disease in type 1 and type 2 diabetes. N Engl J Med..

[17] Roh E, Song DK, Kim M-S (2016). Emerging role of the brain in the homeostatic regulation of energy and glucose metabolism. Exp Mol Med..

[18] Moore B (1906). On the treatment of diabetus mellitus by acid extract of duodenal mucous membrane. Biochem J..

[19] Elrick H, Stimmler L, Hlad CJ, Arai Y (1964). Plasma insulin response to oral and intravenous glucose administration. J Clin Endocrinol Metab.

[20] Mcintyre N, Holdsworth CD, Turner DS (1964). New interpretation of oral glucose tolerance. Lancet (London, England)..

[21] Campbell JE, Drucker DJ (2013). Pharmacology, physiology, and mechanisms of incretin hormone action. Cell Metab.

[22] Bauer PV, Duca FA (2016). Targeting the gastrointestinal tract to treat type 2 diabetes. J Endocrinol.

[23] Mortensen K, Christensen LL, Holst JJ, Orskov C (2003). GLP-1 and GIP are colocalized in a subset of endocrine cells in the small intestine. Regul Pept.

[24] Holst JJ, Orskov C (2004). The incretin approach for diabetes treatment: modulation of islet hormone release by GLP-1 agonism. Diabetes..

[25] Gupta V (2013). Glucagon-like peptide-1 analogues: an overview. Indian J Endocrinol Metab..

[26] Poudyal H (2016). Mechanisms for the cardiovascular effects of glucagon-like peptide-1. Acta Physiol.

[27] Deacon CF, Johnsen AH, Holst JJ (1995). Degradation of glucagon-like peptide-1 by human plasma in vitro yields an N-terminally truncated peptide that is a major endogenous metabolite in vivo. J Clin Endocrinol Metab.

[28] Pabreja K, Mohd MA, Koole C, Wootten D, Furness SGB (2014). Molecular mechanisms underlying physiological and receptor pleiotropic effects mediated by GLP-1R activation. Br J Pharmacol.

[29] Ban K, Noyan-Ashraf MH, Hoefer J, Bolz S-S, Drucker DJ, Husain M (2008). Cardioprotective and vasodilatory actions of glucagon-like peptide 1 receptor are mediated through both glucagon-like peptide 1 receptor-dependent and -independent pathways. Circulation.

[30] Tomas E, Habener JF (2010). Insulin-like actions of glucagon-like peptide-1: a dual receptor hypothesis. Trends Endocrinol Metab.

[31] Liu Z, Stanojevic V, Brindamour LJ, Habener JF (2012). GLP1-derived nonapeptide GLP1(28–36)amide protects pancreatic-cells from glucolipotoxicity. J Endocrinol.

[32] Tomas E, Stanojevic V, Habener JF (2011). GLP-1-derived nonapeptide GLP-1(28–36)amide targets to mitochondria and suppresses glucose production and oxidative stress in isolated mouse hepatocytes. Regul Pept.

[33] Li J, Zheng J, Wang S, Lau HK, Fathi A, Wang Q (2017). Cardiovascular benefits of native GLP-1 and its metabolites: an indicator for GLP-1-therapy strategies. Front Physiol..

[34] Guglielmi V, Sbraccia P (2017). GLP-1 receptor independent pathways: emerging beneficial effects of GLP-1 breakdown products eat weight disord—stud anorexia. Bulim Obes..

[35] Kalra S, Baruah M, Sahay R, Unnikrishnan A, Uppal S, Adetunji O (2016). Glucagon-like peptide-1 receptor agonists in the treatment of type 2 diabetes: past, present, and future. Indian J Endocrinol Metab..

[36] American Diabetes Association (2013). Standards of medical care in diabetes–2013. Diabetes Care.

[37] Lerche S, Soendergaard L, Rungby J, Moeller N, Holst JJ, Schmitz OE (2009). No increased risk of hypoglycaemic episodes during 48 h of subcutaneous glucagon-like-peptide-1 administration in fasting healthy subjects. Clin Endocrinol (Oxf).

[38] Nadkarni P, Chepurny OG, Holz GG (2014). Regulation of glucose homeostasis by GLP-1. Prog Mol Biol Transl Sci..

[39] Tran KL, Park YI, Pandya S, Muliyil NJ, Jensen BD, Huynh K (2017). Overview of glucagon-like peptide-1 receptor agonists for the treatment of patients with type 2 diabetes. Am Health Drug Benefits..

[40] Nauck MA, Vilsbøll T, Gallwitz B, Garber A, Madsbad S (2009). Incretin-based therapies: viewpoints on the way to consensus. Diabetes Care.

[41] Meier JJ (2012). GLP-1 receptor agonists for individualized treatment of type 2 diabetes mellitus. Nat Rev Endocrinol..

[42] Madsbad S (2016). Review of head-to-head comparisons of glucagon-like peptide-1 receptor agonists. Diabetes Obes Metab.

[43] Mikhail N (2006). Exenatide: a novel approach for treatment of type 2 diabetes. South Med J.

[44] Kastin AJ, Akerstrom V (2003). Entry of exendin-4 into brain is rapid but may be limited at high doses. Int J Obes Relat Metab Disord.

[45] Cirincione B, Mager DE (2017). Population pharmacokinetics of exenatide. Br J Clin Pharmacol.

[46] Leon N, LaCoursiere R, Yarosh D, Patel RS (2017). Lixisenatide (Adlyxin): a once-daily incretin mimetic injection for type-2 diabetes. P T..

[47] Okere AN, Montesdeoca J, Glasper A, Diaby V (2017). An Evaluation of the clinical therapeutic effect of lixisenatide in type 2 diabetes patients: a systematic literature review. Curr Diabetes Rev..

[48] Scott LJ (2013). Lixisenatide: a review of its use in patients with type 2 Diabetes mellitus. BioDrugs..

[49] Hunter K, Holscher C (2012). Drugs developed to treat diabetes, liraglutide and lixisenatide, cross the blood brain barrier and enhance neurogenesis. BMC Neurosci..

[50] Christensen M, Miossec P, Larsen BD, Werner U, Knop FK (2014). The design and discovery of lixisenatide for the treatment of type 2 diabetes mellitus. Expert Opin Drug Discov.

[51] Jackson SH, Martin TS, Jones JD, Seal D, Emanuel F (2010). Liraglutide (victoza): the first once-daily incretin mimetic injection for type-2 diabetes. Proc Trans MediMedia.

[52] Ladenheim EE (2015). Liraglutide and obesity: a review of the data so far. Drug Des Devel Ther. Dove Press.

[53] Malm-Erjefält M, Bjørnsdottir I, Vanggaard J, Helleberg H, Larsen U, Oosterhuis B (2010). Metabolism and excretion of the once-daily human glucagon-like peptide-1 analog liraglutide in healthy male subjects and its in vitro degradation by dipeptidyl peptidase IV and neutral endopeptidase. Drug Metab Dispos..

[54] Jacobsen LV, Hindsberger C, Robson R, Zdravkovic M (2009). Effect of renal impairment on the pharmacokinetics of the GLP-1 analogue liraglutide. Br J Clin Pharmacol.

[55] Candeias EM, Sebastião IC, Cardoso SM, Correia SC, Carvalho CI, Plácido AI (2015). Gut–brain connection: the neuroprotective effects of the anti-diabetic drug liraglutide. World J Diabetes..

[56] Hou J, Manaenko A, Hakon J, Hansen-Schwartz J, Tang J, Zhang JH (2012). Liraglutide, a long-acting GLP-1 mimetic, and its metabolite attenuate inflammation after intracerebral hemorrhage. J Cereb Blood Flow Metab..

[57] Weissman PN, Carr MC, Ye J, Cirkel DT, Stewart M, Perry C (2014). HARMONY 4: randomised clinical trial comparing once-weekly albiglutide and insulin glargine in patients with type 2 diabetes inadequately controlled with metformin with or without sulfonylurea. Diabetologia.

[58] Fala L (2015). Tanzeum (Albiglutide): a once-weekly GLP-1 receptor agonist subcutaneous injection approved for the treatment of patients with type 2 diabetes. Am Health Drug Benefits..

[59] Rendell MS (2017). The safety of albiglutide for the treatment of type 2 diabetes. Expert Opin Drug Saf..

[60] Smith LL, Mosley JF, Parke C, Brown J, Barris LS, Phan LD (2016). Dulaglutide (Trulicity): the third once-weekly GLP-1 agonist. Proc Trans MediMedia.

[61] Umpierrez GE, Blevins T, Rosenstock J, Cheng C, Anderson JH, Bastyr EJ (2011). The effects of LY2189265, a long-acting glucagon-like peptide-1 analogue, in a randomized, placebo-controlled, double-blind study of overweight/obese patients with type 2 diabetes: the EGO study. Diabetes Obes Metab.

[62] Kugler AJ, Thiman ML (2018). Efficacy and safety profile of once-weekly dulaglutide in type 2 diabetes: a report on the emerging new data. Diabetes Metab Syndr Obes. Dove Press.

[63] Ferdinand KC, Botros FT, Atisso CM, Sager PT (2016). Cardiovascular safety for once-weekly dulaglutide in type 2 diabetes: a pre-specified meta-analysis of prospectively adjudicated cardiovascular events. Cardiovasc Diabetol..

[64] Sebokova E, Christ AD, Wang H, Sewing S, Dong JZ, Taylor J (2010). Taspoglutide, an analog of human glucagon-like peptide-1 with enhanced stability and in vivo potency. Endocrinology.

[65] Hollander P, Lasko B, Barnett AH, Bengus M, Kanitra L, Pi-Sunyer FX (2013). Effects of taspoglutide on glycemic control and body weight in obese patients with type 2 diabetes (T-emerge 7 study). Obesity..

[66] Rosenstock J, Balas B, Charbonnel B, Bolli GB, Boldrin M, Ratner R (2013). The fate of Taspoglutide, a weekly GLP-1 receptor agonist, versus twice-daily exenatide for type 2 diabetes: the T-emerge 2 trial. Diabetes Care.

[67] Reuters. Roche suspends dosing in diabetes drug trials. https://www.reuters.com/article/us-roche-diabetes/roche-suspends-dosing-in-diabetes-drug-trials-idUSTRE6895FW20100910. Accessed 28 May 2018.

[68] Hjerpsted JB, Flint A, Brooks A, Axelsen MB, Kvist T, Blundell J (2013). Semaglutide improves postprandial glucose and lipid metabolism, and delays first-hour gastric emptying in subjects with obesity. Diabetes Obes Metab..

[69] Nauck MA, Petrie JR, Sesti G, Mannucci E, Courrèges J-P, Lindegaard ML (2016). A phase 2, randomized, dose-finding study of the novel once-weekly human GLP-1 analog, semaglutide, compared with placebo and open-label liraglutide in patients with type 2 diabetes. Diabetes Care.

[70] Davies M, Pieber TR, Hartoft-Nielsen M-L, Hansen OKH, Jabbour S, Rosenstock J (2017). Effect of oral semaglutide compared with placebo and subcutaneous semaglutide on glycemic control in patients with type 2 diabetes. JAMA.

[71] Guja C, Dănciulescu Miulescu R (2017). Semaglutide-the, “new kid on the block” in the field of glucagon-like peptide-1 receptor agonists?. Ann Transl Med..

[72] Trulicity ^®^ (dulaglutide). Full prescribing information. August 2017. http://uspl.lilly.com/trulicity/trulicity.html#pi. Accessed 29 May 2018.

[73] Jensen L, Helleberg H, Roffel A, van Lier JJ, Bjørnsdottir I, Pedersen PJ (2017). Absorption, metabolism and excretion of the GLP-1 analogue semaglutide in humans and nonclinical species. Eur J Pharm Sci.

[74] Nauck MA, Kleine N, Orskov C, Holst JJ, Willms B, Creutzfeldt W (1993). Normalization of fasting hyperglycaemia by exogenous glucagon-like peptide 1 (7–36 amide) in type 2 (non-insulin-dependent) diabetic patients. Diabetologia.

[75] Baggio LL, Drucker DJ (2007). Biology of Incretins: gLP-1 and GIP. Gastroenterology.

[76] Potts JE, Gray LJ, Brady EM, Khunti K, Davies MJ, Bodicoat DH (2015). The effect of glucagon-like peptide 1 receptor agonists on weight loss in type 2 diabetes: a systematic review and mixed treatment comparison meta-analysis. PLoS ONE..

[77] Barragán JM, Rodríguez RE, Blázquez E (1994). Changes in arterial blood pressure and heart rate induced by glucagon-like peptide-1-(7–36) amide in rats. Am J Physiol.

[78] Scheen AJ (2017). GLP-1 receptor agonists and heart failure in diabetes. Diabetes Metab..

[79] Saraiva FK, Sposito AC (2014). Cardiovascular effects of glucagon-like peptide 1 (GLP-1) receptor agonists. Cardiovasc Diabetol..

[80] Ravassa S, Zudaire A, Diez J (2012). GLP-1 and cardioprotection: from bench to bedside. Cardiovasc Res.

[81] Zhao T, Parikh P, Bhashyam S, Bolukoglu H, Poornima I, Shen Y-T (2006). Direct effects of glucagon-like peptide-1 on myocardial contractility and glucose uptake in normal and postischemic isolated rat hearts. J Pharmacol Exp Ther.

[82] Green BD, Hand KV, Dougan JE, McDonnell BM, Cassidy RS, Grieve DJ (2008). GLP-1 and related peptides cause concentration-dependent relaxation of rat aorta through a pathway involving KATP and cAMP. Arch Biochem Biophys.

[83] Golpon HA, Puechner A, Welte T, Wichert PV, Feddersen CO (2001). Vasorelaxant effect of glucagon-like peptide-(7–36)amide and amylin on the pulmonary circulation of the rat. Regul Pept.

[84] Gaspari T, Welungoda I, Widdop RE, Simpson RW, Dear AE (2013). The GLP-1 receptor agonist liraglutide inhibits progression of vascular disease via effects on atherogenesis, plaque stability and endothelial function in an ApoE ^−/−^ mouse model. Diabetes Vasc Dis Res..

[85] Rizzo M, Rizvi AA, Patti AM, Nikolic D, Giglio RV, Castellino G (2016). Liraglutide improves metabolic parameters and carotid intima-media thickness in diabetic patients with the metabolic syndrome: an 18-month prospective study. Cardiovasc Diabetol..

[86] Kumarathurai P, Anholm C, Larsen BS, Olsen RH, Madsbad S, Kristiansen O (2017). Effects of liraglutide on heart rate and heart rate variability: a randomized, double-blind, placebo-controlled crossover study. Diabetes Care..

[87] Kumarathurai P, Anholm C, Nielsen OW, Kristiansen OP, Mølvig J, Madsbad S (2016). Effects of the glucagon-like peptide-1 receptor agonist liraglutide on systolic function in patients with coronary artery disease and type 2 diabetes: a randomized double-blind placebo-controlled crossover study. Cardiovasc Diabetol..

[88] Oyama J-I, Node K (2014). Incretin therapy and heart failure. Circ J.

[89] Nikolaidis LA, Elahi D, Hentosz T, Doverspike A, Huerbin R, Zourelias L (2004). Recombinant glucagon-like peptide-1 increases myocardial glucose uptake and improves left ventricular performance in conscious dogs with pacing-induced dilated cardiomyopathy. Circulation.

[90] Lepore JJ, Olson E, Demopoulos L, Haws T, Fang Z, Barbour AM (2016). Effects of the novel long-acting GLP-1 agonist, albiglutide, on cardiac function, cardiac metabolism, and exercise capacity in patients with chronic heart failure and reduced ejection fraction. JACC Heart Fail..

[91] Scalzo RL, Moreau KL, Ozemek C, Herlache L, McMillin S, Gilligan S (2017). Exenatide improves diastolic function and attenuates arterial stiffness but does not alter exercise capacity in individuals with type 2 diabetes. J Diabetes Complications.

[92] Kim DS, Choi H-I, Wang Y, Luo Y, Hoffer BJ, Greig NH (2017). A new treatment strategy for Parkinson’s disease through the gut–brain axis. Cell Transplant.

[93] Li Y, Perry T, Kindy MS, Harvey BK, Tweedie D, Holloway HW (2009). GLP-1 receptor stimulation preserves primary cortical and dopaminergic neurons in cellular and rodent models of stroke and Parkinsonism. Proc Natl Acad Sci.

[94] Chen F, Wang W, Ding H, Yang Q, Dong Q, Cui M (2016). The glucagon-like peptide-1 receptor agonist exendin-4 ameliorates warfarin-associated hemorrhagic transformation after cerebral ischemia. J Neuroinflammation..

[95] Sato K, Kameda M, Yasuhara T, Agari T, Baba T, Wang F (2013). Neuroprotective effects of liraglutide for stroke model of rats. Int J Mol Sci.

[96] Darsalia V, Klein T, Nyström T, Patrone C (2017). Glucagon-like receptor 1 agonists and DPP-4 inhibitors: anti-diabetic drugs with anti-stroke potential. Neuropharmacology..

[97] Darsalia V, Larsson M, Klein T, Patrone C (2018). The high need for trials assessing functional outcome after stroke rather than stroke prevention with GLP-1 agonists and DPP-4 inhibitors. Cardiovasc Diabetol..

[98] Tanaka A, Node K (2018). Clinical application of glucagon-like peptide-1 receptor agonists in cardiovascular disease: lessons from recent clinical cardiovascular outcomes trials. Cardiovasc Diabetol..

[99] Nauck MA, Meier JJ, Cavender MA, Abd El Aziz M, Drucker DJ (2017). Cardiovascular actions and clinical outcomes with glucagon-like peptide-1 receptor agonists and dipeptidyl peptidase-4 inhibitors. Circulation..

[100] Vergès B, Charbonnel B (2017). After the LEADER trial and SUSTAIN-6, how do we explain the cardiovascular benefits of some GLP-1 receptor agonists?. Diabetes Metab..

[101] Chait A, Bornfeldt KE (2009). Diabetes and atherosclerosis: is there a role for hyperglycemia?. J Lipid Res..

[102] Shah MS, Brownlee M (2016). Molecular and cellular mechanisms of cardiovascular disorders in diabetes. Circ Res.

[103] UK Prospective Diabetes Study (1998). Intensive blood-glucose control with sulphonylureas or insulin compared with conventional treatment and risk of complications in patients with type 2 diabetes (UKPDS 33). Lancet..

[104] Patel A, MacMahon S, Chalmers J, Neal B, Billot L, ADVANCE Collaborative Group (2008). Intensive blood glucose control and vascular outcomes in patients with type 2 diabetes. N Engl J Med..

[105] Del Olmo-Garcia MI, Merino-Torres JF (2018). GLP-1 receptor agonists and cardiovascular disease in patients with type 2 diabetes. J Diabetes Res..

[106] Wang B, Zhong J, Lin H, Zhao Z, Yan Z, He H (2013). Blood pressure-lowering effects of GLP-1 receptor agonists exenatide and liraglutide: a meta-analysis of clinical trials. Diabetes Obes Metab.

[107] Sun F, Wu S, Guo S, Yu K, Yang Z, Li L (2015). Impact of GLP-1 receptor agonists on blood pressure, heart rate and hypertension among patients with type 2 diabetes: a systematic review and network meta-analysis. Diabetes Res Clin Pract.

[108] Garber AJ (2012). Postprandial dysmetabolism and the heart. Heart Fail Clin..

[109] Ginsberg HN, Illingworth DR (2001). Postprandial dyslipidemia: an atherogenic disorder common in patients with diabetes mellitus. Am J Cardiol.

[110] Pang J, Chan DC, Barrett PHR, Watts GF (2012). Postprandial dyslipidaemia and diabetes. Curr Opin Lipidol.

[111] Farr S, Taher J, Adeli K (2014). Glucagon-like peptide-1 as a key regulator of lipid and lipoprotein metabolism in fasting and postprandial states. Cardiovasc Hematol Disord Drug Targets.

[112] Voukali M, Kastrinelli I, Stragalinou S, Tasiopoulou D, Paraskevopoulou P, Katsilambros N (2014). Study of postprandial lipaemia in type 2 diabetes mellitus: exenatide versus liraglutide. J Diabetes Res..

[113] Schwartz EA, Koska J, Mullin MP, Syoufi I, Schwenke DC, Reaven PD (2010). Exenatide suppresses postprandial elevations in lipids and lipoproteins in individuals with impaired glucose tolerance and recent onset type 2 diabetes mellitus. Atherosclerosis..

[114] Gaiz A, Mosawy S, Colson N, Singh I (2017). Thrombotic and cardiovascular risks in type two diabetes. Role of platelet hyperactivity. Biomed Pharmacother..

[115] Cameron-Vendrig A, Reheman A, Siraj MA, Xu XR, Wang Y, Lei X (2016). Glucagon-like peptide 1 receptor activation attenuates platelet aggregation and thrombosis. Diabetes.

[116] Jia G, Aroor AR, Sowers JR (2016). Glucagon-like peptide 1 receptor activation and platelet function: beyond glycemic control. Diabetes.

[117] Hanefeld M, Frier BM, Pistrosch F (2016). Hypoglycemia and cardiovascular risk: is there a major Link?. Diabetes Care.

[118] Ginsberg HN (2000). Insulin resistance and cardiovascular disease. J Clin Invest..

[119] MacDonald PE, El-Kholy W, Riedel MJ, Salapatek AMF, Light PE, Wheeler MB (2002). The multiple actions of GLP-1 on the process of glucose-stimulated insulin secretion. Diabetes..

[120] Sena CM, Pereira AM, Seiça R (2013). Endothelial dysfunction—a major mediator of diabetic vascular disease. Biochim Biophys Acta.

[121] Gimbrone MA, García-Cardeña G (2016). Endothelial cell dysfunction and the pathobiology of atherosclerosis. Circ Res.

[122] Nyström T, Gutniak MK, Zhang Q, Zhang F, Holst JJ, Ahrén B (2004). Effects of glucagon-like peptide-1 on endothelial function in type 2 diabetes patients with stable coronary artery disease. Am J Physiol Metab..

[123] Koska J, Sands M, Burciu C, D’Souza KM, Raravikar K, Liu J (2015). Exenatide protects against glucose- and lipid-induced endothelial dysfunction: evidence for direct vasodilation effect of GLP-1 receptor agonists in humans. Diabetes.

[124] Gaspari T, HongBin Liu H, Welungoda I, Yunshan HuY, Widdop RE, Knudsen LB (2011). A GLP-1 receptor agonist liraglutide inhibits endothelial cell dysfunction and vascular adhesion molecule expression in an ApoE ^−/−^ mouse model. Diabetes Vasc Dis Res..

[125] Irace C, De Luca S, Shehaj E, Carallo C, Loprete A, Scavelli F (2013). Exenatide improves endothelial function assessed by flow mediated dilation technique in subjects with type 2 diabetes: results from an observational research. Diabetes Vasc Dis Res..

[126] Hopkins ND, Cuthbertson DJ, Kemp GJ, Pugh C, Green DJ, Cable NT (2013). Effects of 6 months glucagon-like peptide-1 receptor agonist treatment on endothelial function in type 2 diabetes mellitus patients. Diabetes Obes Metab.

[127] Quinaglia T, Matos-Souza JR, Feinstein SB, Sposito AC (2015). Flow-mediated dilation: an evolving method. Atherosclerosis..

[128] Ha SJ, Kim W, Woo JS, Kim JB, Kim SJ, Kim W-S (2012). Preventive effects of exenatide on endothelial dysfunction induced by ischemia–reperfusion injury via KATP channels. Arterioscler Thromb Vasc Biol.

[129] Lønborg J, Vejlstrup N, Kelbæk H, Bøtker HE, Kim WY, Mathiasen AB (2012). Exenatide reduces reperfusion injury in patients with ST-segment elevation myocardial infarction. Eur Heart J.

[130] Chen WR, Chen YD, Tian F, Yang N, Cheng LQ, Hu SY (2016). Effects of liraglutide on reperfusion injury in patients with st-segment-elevation myocardial infarction. Circ Cardiovasc Imaging.

[131] Chen WR, Hu SY, Chen YD, Zhang Y, Qian G, Wang J (2015). Effects of liraglutide on left ventricular function in patients with ST-segment elevation myocardial infarction undergoing primary percutaneous coronary intervention. Am Heart J.

[132] Wei R, Ma S, Wang C, Ke J, Yang J, Li W (2016). Exenatide exerts direct protective effects on endothelial cells through the AMPK/Akt/eNOS pathway in a GLP-1 receptor-dependent manner. Am J Physiol Metab..

[133] Ban K, Kim K-H, Cho C-K, Sauvé M, Diamandis EP, Backx PH (2010). Glucagon-like peptide (GLP)-1(9–36)amide-mediated cytoprotection is blocked by exendin(9–39) yet does not require the known GLP-1 receptor. Endocrinology.

[134] Sonne DP, Engstrøm T, Treiman M (2008). Protective effects of GLP-1 analogues exendin-4 and GLP-1(9–36) amide against ischemia-reperfusion injury in rat heart. Regul Pept.

[135] Szeto HH (2006). Cell-permeable, mitochondrial-targeted, peptide antioxidants. AAPS J.

[136] Chatre L, Matheson LA, Jack AS, Hanton SL, Brandizzi F (2009). Efficient mitochondrial targeting relies on co-operation of multiple protein signals in plants. J Exp Bot.

[137] Federal Drug Administration (FDA). Guidance for industry diabetes mellitus—evaluating cardiovascular risk in new antidiabetic therapies to treat type 2 diabetes. 2008. http://www.fda.gov/downloads/Drugs/GuidanceComplianceRegulatoryInformation/Guidances/u.

[138] Marso SP, Daniels GH, Brown-Frandsen K, Kristensen P, Mann JFE, Nauck MA (2016). Liraglutide and cardiovascular outcomes in type 2 diabetes. N Engl J Med.

[139] Best JH, Hoogwerf BJ, Herman WH, Pelletier EM, Smith DB, Wenten M (2011). Risk of cardiovascular disease events in patients with type 2 diabetes prescribed the glucagon-like peptide 1 (GLP-1) receptor agonist exenatide twice daily or other glucose-lowering therapies: a retrospective analysis of the lifelink DATABASE. Diabetes Care.

[140] Holman RR, Bethel MA, Mentz RJ, Thompson VP, Lokhnygina Y, Buse JB (2017). Effects of once-weekly exenatide on cardiovascular outcomes in type 2 diabetes. N Engl J Med.

[141] Pfeffer MA, Claggett B, Diaz R, Dickstein K, Gerstein HC, Køber LV (2015). Lixisenatide in patients with type 2 diabetes and acute coronary syndrome. N Engl J Med.

[142] Gerstein HC, Miller ME, Byington RP, Goff DC, Bigger JT, Buse JB, Action to Control Cardiovascular Risk in Diabetes Study Group (2008). Effects of intensive glucose lowering in type 2 diabetes. N Engl J Med..

[143] Hayward RA, Reaven PD, Emanuele NV, VADT Investigators (2015). Follow-up of glycemic control and cardiovascular outcomes in type 2 diabetes. N Engl J Med..

[144] Zinman B, Marso SP, Christiansen E, et al. Severe hypoglycemia, cardiovascular outcomes and death—the LEADER experience. Oral presentation 359-OR. In: 77th Annual Meeting of the American Diabetes Association (ADA), San Diego, USA; 9–13 June 2017.

[145] Vilsbøll T, Bain SC, Leiter LA, Lingvay I, Matthews D, Simó R (2017). Semaglutide, reduction in HbA _1c_ and the risk of diabetic retinopathy. Diabetes Obes Metab.

[146] Hernandez AF, Green JB, Janmohamed S, D’Agostino RB, Granger CB, Jones NP (2018). Albiglutide and cardiovascular outcomes in patients with type 2 diabetes and cardiovascular disease (Harmony Outcomes): a double-blind, randomised placebo-controlled trial. Lancet (London, England)..

[147] Gerstein HC, Colhoun HM, Dagenais GR, Diaz R, Lakshmanan M, Pais P (2018). Design and baseline characteristics of participants in the researching cardiovascular events with a weekly INcretin in diabetes (REWIND) trial on the cardiovascular effects of dulaglutide. Diabetes Obes Metab.

[148] Cefalu WT, Kaul S, Gerstein HC, Holman RR, Zinman B, Skyler JS (2018). Cardiovascular outcomes trials in type 2 diabetes: where do we go from here? reflections from a diabetes care editors’ expert forum. Diabetes Care.

[149] Marso SP, Bain SC, Consoli A, Eliaschewitz FG, Jódar E, Leiter LA (2016). Semaglutide and cardiovascular outcomes in patients with type 2 diabetes. N Engl J Med.

[150] Margulies KB, Hernandez AF, Redfield MM, Givertz MM, Oliveira GH, Cole R (2016). Effects of liraglutide on clinical stability among patients with advanced heart failure and reduced ejection fraction. JAMA.

[151] LYDIA. Effects of liraglutide in young adult a with type 2 diabetes. https://clinicaltrials.gov/ct2/show/NCT02043054.

[152] Bain SC, Mosenzon O, Arechavaleta R, Bogdański P, Comlekci A, Consoli A (2018). Cardiovascular safety of oral semaglutide in patients with type 2 diabetes: rationale, design and patient baseline characteristics for the PIONEER 6 trial. Diabetes Obes Metab..

[153] Gallwitz B (2018). The cardiovascular benefits associated with the use of sodium-glucose cotransporter 2 inhibitors—real-world data. Eur Endocrinol..

[154] Zheng SL, Roddick AJ, Aghar-Jaffar R, Shun-Shin MJ, Francis D, Oliver N (2018). Association between use of sodium-glucose cotransporter 2 inhibitors, glucagon-like peptide 1 agonists, and dipeptidyl peptidase 4 inhibitors with all-cause mortality in patients with type 2 diabetes: a systematic review and meta-analysis. JAMA.

[155] Goncalves E, Bell DSH (2018). Combination treatment of SGLT2 inhibitors and GLP-1 receptor agonists: symbiotic effects on metabolism and cardiorenal risk. Diabetes Ther..

[156] Darsalia V, Larsson M, Nathanson D, Klein T, Nyström T, Patrone C (2015). Glucagon-like receptor 1 agonists and DPP-4 inhibitors: potential therapies for the treatment of stroke. J Cereb Blood Flow Metab.

