# Correction to: GLP-1RAs in type 2 diabetes: mechanisms that underlie cardiovascular effects and overview of cardiovascular outcome data

**DOI:** 10.1186/s12933-019-0825-1

**Published:** 2019-03-01

**Authors:** Andrei C. Sposito, Otávio Berwanger, Luiz Sérgio F. de Carvalho, José Francisco Kerr Saraiva

**Affiliations:** 1 Atherosclerosis and Vascular Biology Laboratory (AtheroLab), Cardiology Division, Faculty of Medical Sciences, State University of Campinas (Unicamp), Campinas, Sao Paulo 13084-971 Brazil; 2 Academic Research Organization (ARO), Albert Einstein Hospital, Av. Albert Einstein 627, Sao Paulo, SP 05651-90 Brazil; 3 Cardiology Division, Pontifical Catholic University of Campinas Medicine School, Rua Engenheiro Carlos Stevenson 560, Campinas, Sao Paulo 13092-132 Brazil

## Correction to: Cardiovascular Diabetology (2018) 17:157 10.1186/s12933-018-0800-2

Following publication of the original article [1], based on the authors review, the GLP1 receptor agonists in type 2 diabetes published in Cardiovascular Diabetology, a meta-analysis of GLP-1 and non-GLP-1 based therapies was performed on cardiovascular outcomes. Unfortunately, as the authors later realized, there was an error in the equation used to calculate the relative risks. Instead of subtracting the number of events from the total number of patients in the sample, these values were added which generated a similar increase in all studies. They reframed the analyzes and found small differences, which were relatively proportional to each study. There was no change in the significance of the analyzes; i.e. all data that were initially statistically significant remained significant and vice versa. The changes have been corrected in the new figures (Figs. 1, 2, Additional file 1) presented below. There was no compromise of any of the conclusions of the text and, therefore, no change was made in the manuscript.

**Fig. 1:**
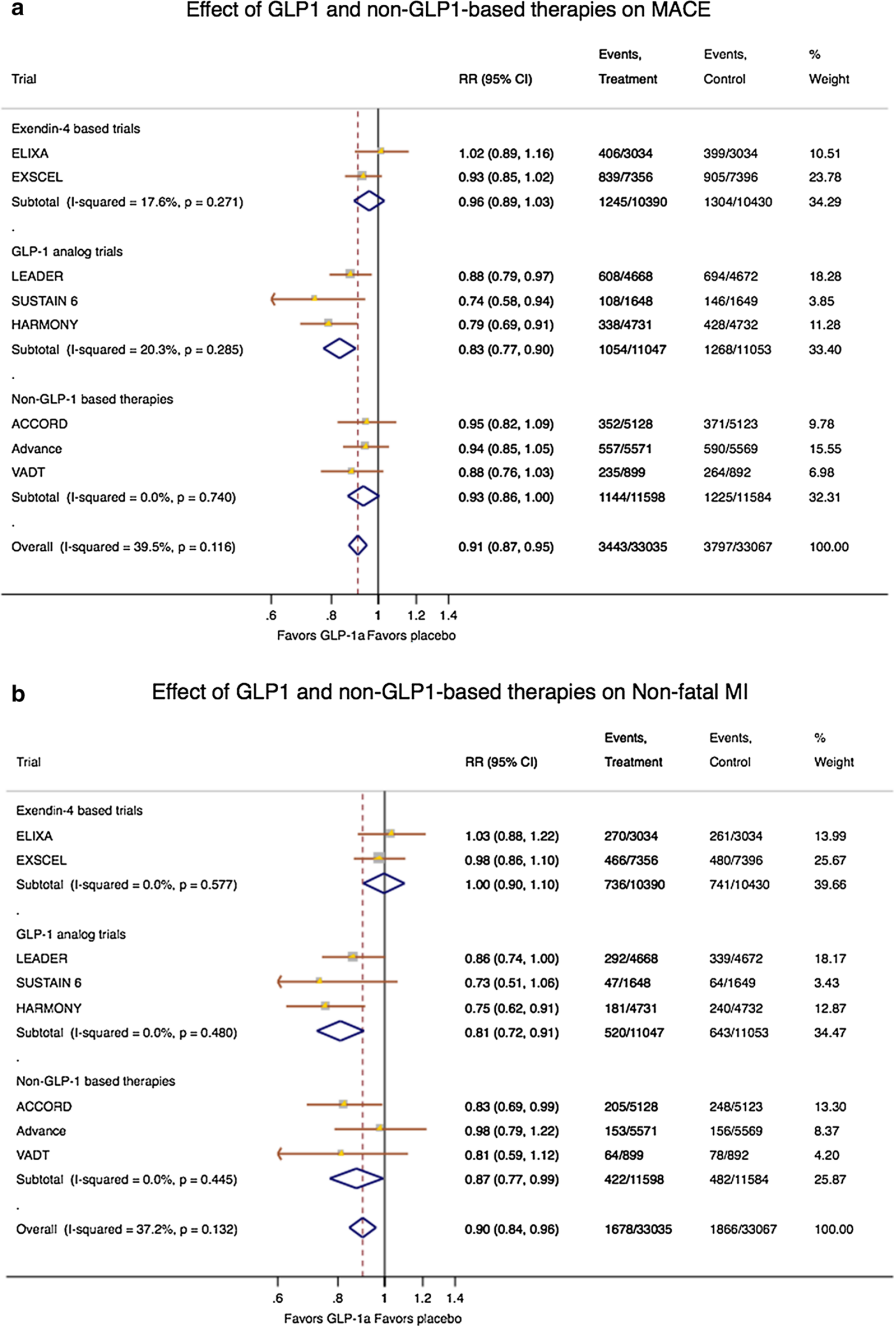
Forest plots showing the effects of GLP-1- and non-GLP-1-based therapies on **a** therapies on a MACE, **b** non-fatal MI

**Fig. 2:**
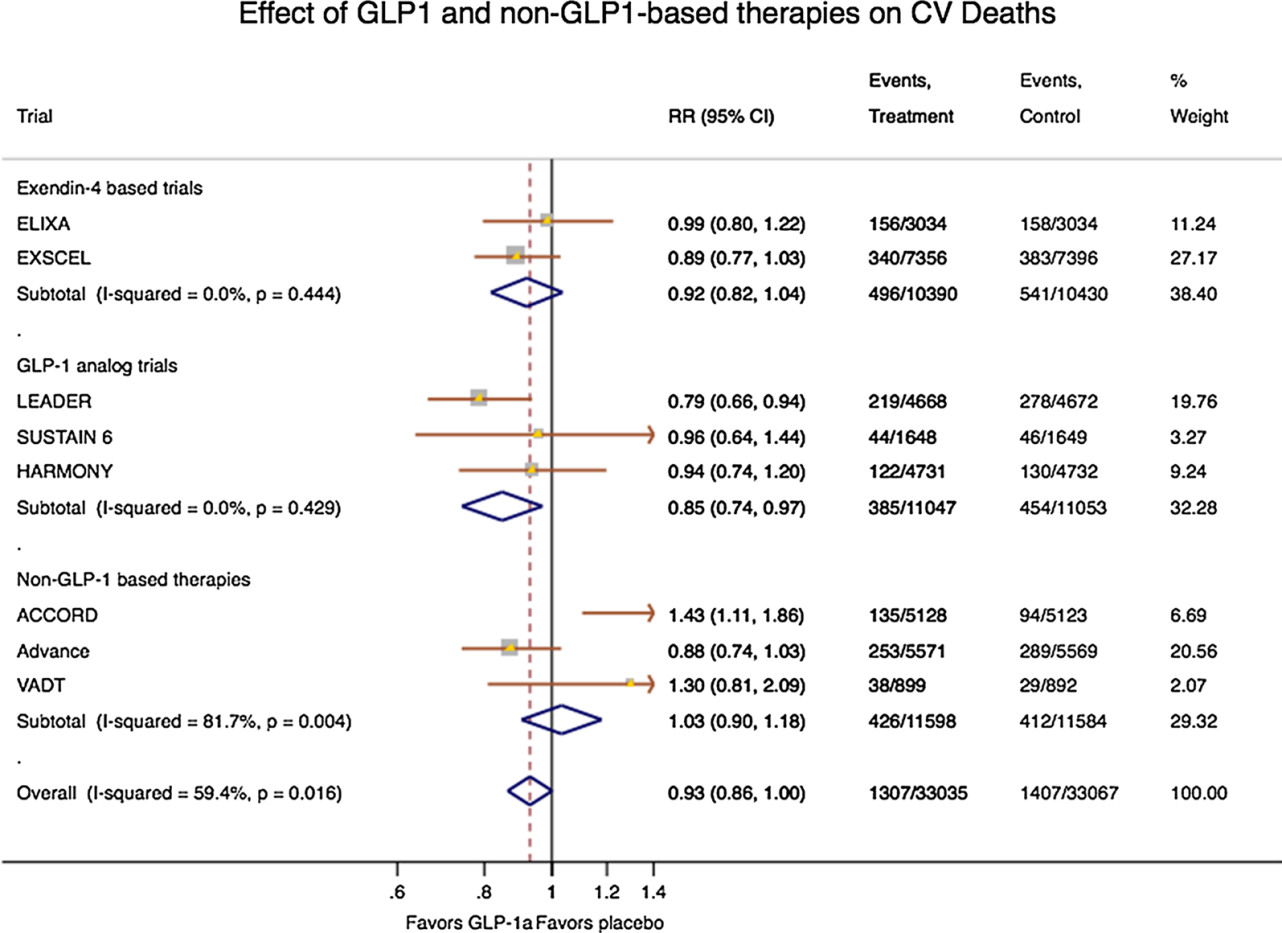
Forest plots showing the effects of GLP-1- and non-GLP-1-based therapies on cardiovascular death

## Additional files

**Additional file 1: Table S1.** Risk of Bias of Individual Randomized Controlled Trials. **Table S2.** Egger Bias Analysis. **Table S3.** Differences in p-values for all trials combined according to the choice of fixed or random methods. **Figure S1.** Funnel plots for myocardial infarction. **Figure S2.** Funnel plots for MACE. **Figure S3.** Funnel plots for Death due to heart failure. **Figure S4.** Funnel plots for CV death. **Figure S5.** Funnel plots for All-cause death.
