# Supplementary material for: Correction to: GLP-1RAs in type 2 diabetes: mechanisms that underlie cardiovascular effects and overview of cardiovascular outcome data
Source: Cardiovasc Diabetol. 2019 Mar 1;18:23. doi: 10.1186/s12933-019-0825-1 (PMC6396451; doi:10.1186/s12933-019-0825-1)

**Table S1: Risk of Bias of Individual Randomized Controlled Trials.**

| **Study**  **(first author)** | **Multicenter trial** | **Adequate Sequence Generation** | **Allocation**  **concealment** | **Blinding** | | | **Incomplete**  **Data**  **Outcome**  **Addressed?** | **Selective Outcome Reporting** | **Free of Other Bias** |
| --- | --- | --- | --- | --- | --- | --- | --- | --- | --- |
|  |  |  |  | **Patient** | **Physician** | **Adjudication of outcomes** |  |  |  |
| ELIXA | Yes | Yes | Yes | Yes | Yes | Yes | Yes | No | Yes |
| LEADER | Yes | Yes | Yes | Yes | Yes | Yes | Yes | No | Yes |
| SUSTAIN 6 | Yes | Yes | Yes | Yes | Yes | Yes | Yes | No | Yes |
| EXSCEL | Yes | Yes | Yes | Yes | Yes | Yes | Yes | No | Yes |
| HARMONY | Yes | Yes | Yes | Yes | Yes | Yes | Yes | No | Yes |
| ACCORD | Yes | Yes | Yes | Yes | Yes | Yes | Yes | No | Yes |
| ADVANCE | Yes | Yes | Yes | Yes | Yes | Yes | Yes | No | Yes |
| VADT | Yes | Yes | Yes | Yes | Yes | Yes | Yes | No | Yes |

**Table S2: Egger Bias Analysis.**

| **Endpoint** | **Egger 2-tailed p-value** |
| --- | --- |
| Myocardial infarction | 0·24 |
| MACE | 0·30 |
| Death due to heart failure | 0·81 |
| CV death | 0·12 |
| All-cause death | 0·38 |

**Table S3: Differences in p-values for all trials combined according to the choice of fixed or random methods.**

| **Endpoint** | **Fixed** | **Random** |
| --- | --- | --- |
| Myocardial infarction | 0·001 | 0·001 |
| MACE | <0·001 | <0·001 |
| Death due to heart failure | 0·526 | 0·562 |
| CV death | 0·054 | 0·102 |
| All-cause death | 0·060 | 0·088 |

**Figure S1: Funnel plots for myocardial infarction.**


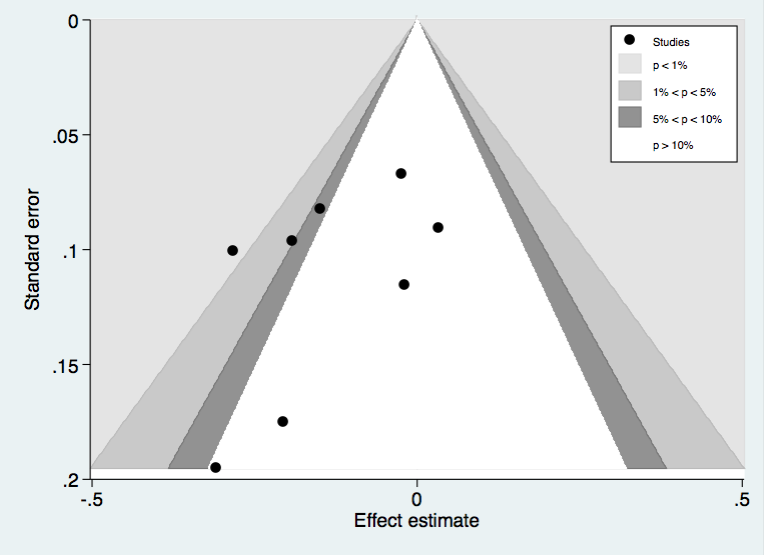


**Figure S2: Funnel plots for MACE.**


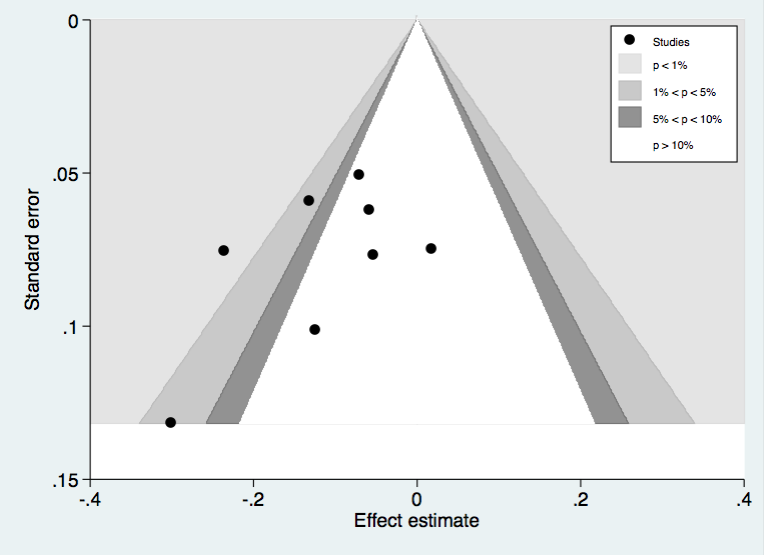


**Figure S3: Funnel plots for Death due to heart failure.**


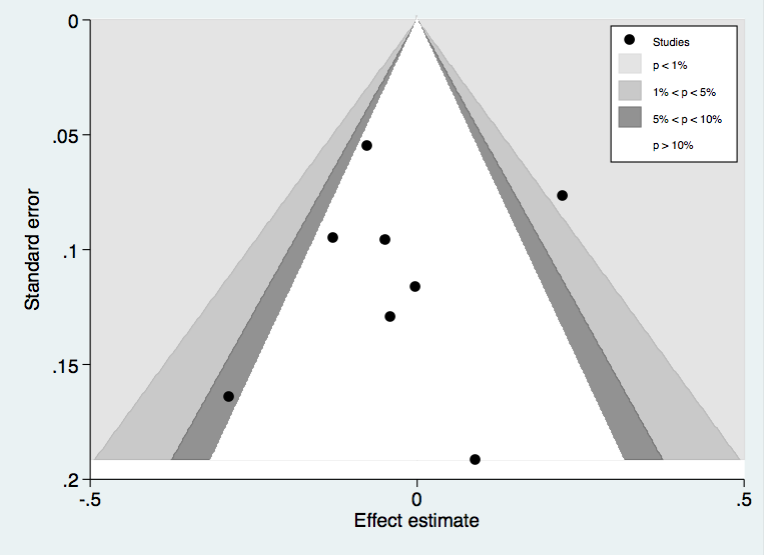


**Figure S4: Funnel plots for CV death.**


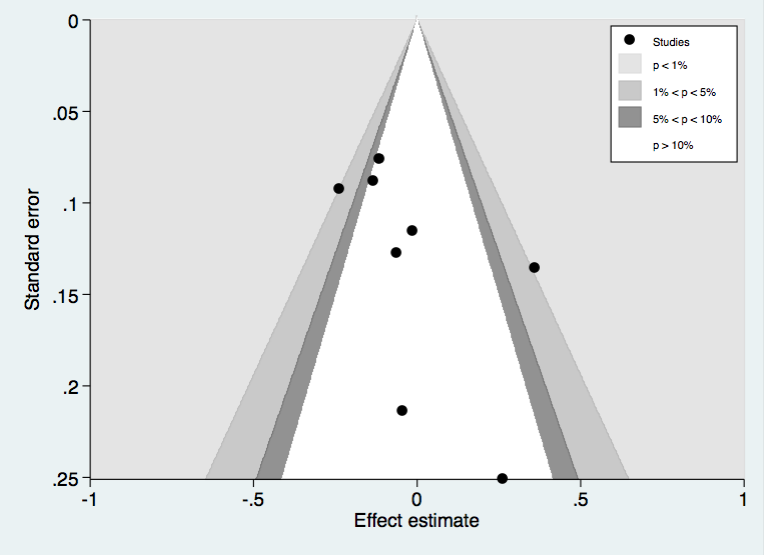


**Figure S5: Funnel plots for All-cause death.**


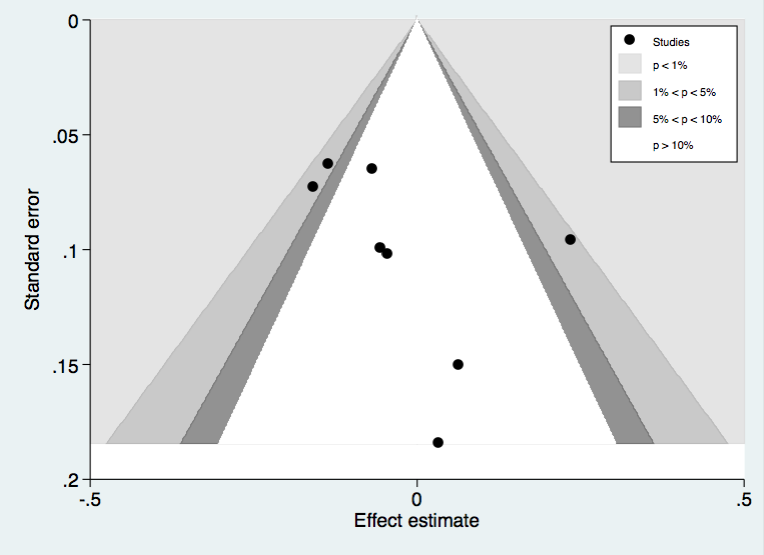

Supplement: Supplementary file 1 — Additional file 1: Table S1. Risk of Bias of Individual Randomized Controlled Trials. Table S2. Egger Bias Analysis. Table S3. Differences in p-values for all trials combined according to the choice of fixed or random methods. Figure S1. Funnel plots for myocardial infarction. Figure S2. Funnel plots for MACE. Figure S3. Funnel plots for Death due to heart failure. Figure S4. Funnel plots for CV death. Figure S5. Funnel plots for All-cause death. [file 12933_2019_825_MOESM1_ESM.docx]
